# Immunofibrogenic Gene Expression Patterns in Tanzanian Children with Ocular *Chlamydia trachomatis* Infection, Active Trachoma and Scarring: Baseline Results of a 4-Year Longitudinal Study

**DOI:** 10.3389/fcimb.2017.00406

**Published:** 2017-09-15

**Authors:** Athumani M. Ramadhani, Tamsyn Derrick, David Macleod, Patrick Massae, Tara Mtuy, David Jeffries, Chrissy H. Roberts, Robin L. Bailey, David C. W. Mabey, Martin J. Holland, Matthew J. Burton

**Affiliations:** ^1^Clinical Research Department, Faculty of Infectious and Tropical Diseases, London School of Hygiene and Tropical Medicine London, United Kingdom; ^2^Kilimanjaro Christian Medical Centre Moshi, Tanzania; ^3^Department of Infectious Disease Epidemiology, Faculty of Epidemiology and Population Health, London School of Hygiene and Tropical Medicine London, United Kingdom; ^4^Support Services (Statistics), Medical Research Council Unit The Gambia Fajara, Gambia

**Keywords:** trachoma, *Chlamydia trachomatis*, gene expression, mass azithromycin administration, longitudinal study, active trachoma, conjunctival scarring

## Abstract

Trachoma, caused by *Chlamydia trachomatis*, is the world's leading infectious cause of blindness and remains a significant public health problem. Much of trachomatous disease pathology is thought to be caused indirectly by host cellular and immune responses, however the immune response during active trachoma and how this initiates progressive scarring is not clearly understood. Defining protective vs. pathogenic immune response to *C. trachomatis* is important for vaccine design and evaluation. This study reports the baseline results of a longitudinal cohort of Tanzanian children, who were monitored for 4 years in order to determine the immunofibrogenic and infectious correlates of progressive scarring trachoma. In this cohort baseline, 506 children aged 6–10 years were assessed for clinical signs, infection status and the expression of 91 genes of interest prior to mass azithromycin administration for trachoma control. *C. trachomatis* was detected using droplet digital PCR and gene expression was measured using quantitative real-time PCR. The prevalence of follicles, papillary inflammation and scarring were 33.6, 31.6, and 28.5%, respectively. *C. trachomatis* was detected in 78/506 (15.4%) individuals, 62/78 of whom also had follicles. *C. trachomatis* infection was associated with a strong upregulation of *IFNG* and *IL22*, the enrichment of Th1 and NK cell pathways and Th17 cell-associated cytokines. In individuals with inflammation in the absence of infection the *IFNG*/*IL22* and NK cell response was reduced, however, pro-inflammatory, growth and matrix factors remained upregulated and mucins were downregulated. Our data suggest that, strong *IFNG*/*IL22* responses, probably related to Th1 and NK cell involvement, is important for clearance of *C. trachomatis* and that the residual pro-inflammatory and pro-fibrotic phenotype that persists after infection might contribute to pathological scarring. Interestingly, females appear more susceptible to developing papillary inflammation and scarring than males, even at this young age, despite comparable levels of *C. trachomatis* infection. Females also had increased expression of a number of IFNγ pathway related genes relative to males, suggesting that overexpression of this pathway in response to infection might contribute to more severe scarring. Longitudinal investigation of these factors will reveal their relative contributions to protection from *C. trachomatis* infection and development of scarring complications.

## Introduction

Trachoma, a Neglected Tropical Disease caused by the bacterium *Chlamydia trachomatis*, remains the leading infectious cause of blindness worldwide. It is characterized by repeated conjunctival infection initiated early in childhood, triggering inflammation (*trachomatous inflammation—intense* and *trachomatous inflammation—follicular*) that drives scarring and trichiasis (in-turned eyelashes) and eventually blinding corneal opacification (Hu et al., 2013a; Taylor et al., 2014; Ramadhani et al., 2016a). The burden of this disease is high; current estimates indicate 200 million people live in trachoma endemic areas in 42 countries (World Health Organization, 2016). Approximately 1.9 million people are visually impaired or irreversibly blind from trachoma (Bourne et al., 2013). To meet this public health challenge, the WHO-led Global Alliance for the Elimination of Trachoma recommends the implementation of the SAFE strategy which tackles the disease at different stages: **S**urgery to correct trichiasis, **A**ntibiotics to treat chlamydial infection and **F**acial cleanliness and **E**nvironmental improvements to suppress transmission of infection (Taylor et al., 2014).

Our understanding of this disease process is only partial (Hu et al., 2013a). There are few detailed long-term longitudinal studies that investigate the risk factors for and pathophysiology of scarring trachoma (Dawson et al., 1990; West et al., 2001; Wolle et al., 2009a,b; Burton et al., 2015). These studies have consistently found incident and progressive scarring to be strongly associated with clinically apparent conjunctival inflammation (Ramadhani et al., 2016a). However, only one study has prospectively examined the relationship between *C. trachomatis* infection and the development of incident scarring in younger people; this found that the detection of infection and/or severe inflammation on multiple occasions was associated with increased risk of new scarring (Wolle et al., 2009b). The relationship between chlamydial infection and progression of previously established scarring in adults has been prospectively examined in two longitudinal cohorts; neither found an association (Burton et al., 2015). This raises the possibility that progressive scarring may not be entirely dependent on continual re-exposure to *C. trachomatis*. It is possible that repeated chlamydial infection results in long-term physiological changes in conjunctival tissue responsiveness, such that other pro-inflammatory factors may stimulate on-going fibrotic responses (Kechagia et al., 2016). For trachoma control programmes, this raises the possibility that scarring may continue to progress after ocular chlamydial infection has been eliminated from the population. The implication of this is that services for managing incident trichiasis may be needed for many years.

Previously, we and others have explored the immunological correlates of the different stages of trachoma mostly through cross-sectional studies using a range of methodologies, including gene expression and protein analysis, immunohistochemistry and genome wide association studies (Bobo et al., 1996; Burton et al., 2004, 2011b, 2015; Skwor et al., 2008; Holland et al., 2010; Natividad et al., 2010; Derrick et al., 2013, 2016a,b; Hu et al., 2013a, 2016; Roberts et al., 2015). These studies have shown an increase in pro-inflammatory and matrix factors (*IL1B* (interleukin 1 beta), *TNF* (tumor necrosis factor), *S100A7* (psoriasin), *IL17A, IFNG* (interferon gamma), perforin, *IL12, IL10, CXCL5, CTGF* (connective tissue growth factor) and *MMP9* (matrix metalloproteinase 9) in individuals with active trachoma and/or chlamydial infection, indicating the involvement of a type 1 T helper (Th1) cell response, NK cell cytotoxicity and potentially Th17 cells. In individuals with trachomatous scarring and/or trichiasis, factors involved in innate pro-inflammatory responses and matrix remodeling (*IL1B, CXCL5, S100A7, CTGF, MMP7*, and *MMP9*) were upregulated.

To understand the immunofibrogenic correlates of progressive conjunctival scarring we conducted a long-term cohort study. Here we present the baseline findings for a large panel of factors, to define those to be examined in the prospective study.

## Methods

### Ethics statement

This study was reviewed and approved by the Tanzanian National Institute for Medical Research Ethics Committee, the Kilimanjaro Christian Medical Centre Ethics Committee, and the London School of Hygiene and Tropical Medicine Ethics Committee. It adhered to the tenets of the Declaration of Helsinki.

### Study population

The study was conducted in three adjacent trachoma endemic villages in Kilimanjaro and Arusha regions, Northern Tanzania. The villages are relatively remote, geographically neighbors and have similar patterns of life and traditions. This area is predominantly inhabited by people of the Maasai tribe. Pastoral activities are the main occupation. The area is dry for much of the year, except for the rainy season (February to May). Water supply is therefore limited, and largely depends on a long-distance water pipe scheme from Mount Kilimanjaro. Family units are organized in Bomas, with living huts arranged in a circle around a central animal enclosure, which is often characterized by a high density of flies.

In January 2012, we recruited a cohort of children aged 6–10 years from these villages. The cohort has subsequently been followed-up every 3 months for 4 years to investigate the pathogenesis of conjunctival scarring. All children, aged 6–10 years, who were normally resident in one of the three villages, were eligible for inclusion. We chose this restricted age group as we considered that they were more likely to show evidence of incident or progressive conjunctival scarring during the 4 years of the study. At the outset community meetings were held to introduce the study. Each household was then visited to meet the parents or legal guardians of children eligible for enrolment. A field worker explained the nature of the study in detail in either Swahili or Maasai language. There was an opportunity to discuss and ask questions. Finally, if the parent or guardian agreed to allow the child to be enrolled into the study this was documented on a consent form in Kiswahili, and witnessed by a third person.

### Clinical assessments and sample collection

The left eye of each child was examined by an ophthalmic nurse experienced in grading trachoma. The examinations were all performed under standardized conditions, using x2.5 loupes and a bright touch. The conjunctiva was anesthetized with a drop of preservative-free proxymetacaine hydrochloride 0.5%w/v (Minims®, Chauvin Pharmaceuticals Ltd, Surrey, UK). The eyelid was everted, examined and photographed (Nikon D90 with 105 mm Macro lens). Two conjunctival swab samples (Dacron polyester, Puritan Medical Products Company, Maine, USA) were collected for *C. trachomatis* detection and gene expression analysis. The swabs were passed across the upper tarsal conjunctiva four times, with a quarter turn between each pass. The first swab was placed directly into a tube containing RNAlater (Thermo Fisher Scientific, Massachusetts, USA) and the second into a dry tube. The samples were placed into a cool box. Later the same day the dry swab samples were stored directly at −80°C and the RNAlater samples kept at 4–8°C overnight and then stored at −80°C. Air control swabs were collected after every 50 samples by passing a swab 10 cm from a participant's everted eye, these were labeled and processed identically to participant samples.

Clinical signs were graded using the 1981 WHO Detailed Trachoma Grading System (FPC) (Dawson et al., 1981). This sub-divides the features into several four-point severity scales: follicles (F), papillary inflammation (P), and conjunctival scarring (C). This system corresponds to the WHO Simplified Trachoma Grading System in the following way: *Trachomatous inflammation-Follicular* (TF) is equivalent to F2/F3 and *Trachomatous inflammation-Intense* (TI) is equivalent to P3 (Thylefors et al., 1987). For the purpose of this study, we consider that both P2 and P3 represent clinically significant papillary inflammation, and refer to this as “TP” (Burton et al., 2015). We followed the widely used definition of “Clinically Active Trachoma”: TF and/or TI. The digital photographs were graded by an ophthalmologist experienced in trachoma assessment, using the FPC system, supplemented by a previously described system for fine grading of conjunctival scarring, that quantifies the extent of the conjunctiva involved (Hu et al., 2011). The ophthalmologist was masked to the infection status.

Extensive public health education about trachoma was provided to the community through village level meetings and during the house-to-house visits, including the importance of face washing for children and environmental improvements. Trichiasis surgery was provided free of charge within the community. Subsequently, all residents of the three villages have been offered three rounds of annual mass antibiotic treatment with oral azithromycin (and topical tetracycline ointment for infants under 6 months and pregnant women), during the course of the longitudinal study.

### *C. trachomatis* detection

To detect *C. trachomatis*, we extracted DNA from swab samples stored in dry tubes using the PowerSoil DNA Isolation Kit (Mo Bio Laboratories, California, USA), according to the manufacturer's instructions. The cells attached to the swabs were initially disrupted by bead beating to release their contents. *C. trachomatis* DNA was detected using a previously described droplet digital PCR assay (Roberts et al., 2013). All samples were tested for the *C. trachomatis* plasmid and the human gene *RPP30*. Samples in which *C. trachomatis* plasmid was detected were retested for *C. trachomatis omcB* (a single-copy gene on the chlamydial chromosome). Five microliters of template DNA was added per reaction. PCR reaction conditions were as follows: 95°C for 10 min, then 40 cycles of 95°C for 10 s and 60°C for 30 s and finally 98°C for 12 min. Droplets were then examined for fluorescence on a QX100 Droplet Reader (Bio-Rad, UK), providing a quantitative result.

### Human gene expression analysis

Total RNA was extracted from the swab stored in RNALater using a DNA/RNA Purification Kit (Norgen Biotek Corp, Canada), following the manufacturer's instructions. RNA was reverse transcribed using the SuperScript® VILO® cDNA Synthesis Kit (Life Technologies). Quantitative real-time PCR was performed to measure the relative abundance of a panel of human transcripts, using the TaqMan® Microfluidic 384-well Low-Density Array (TLDA) (Life Technologies) on a ViiA7 real-time PCR instrument (Thermo Fisher Scientific, Massachusetts, USA). These cards have separate channels for eight samples, each containing 48 wells, pre-printed with the assay primers and probes. We used two different TLDA designs and measured the expression of 91 different genes of interest for each sample. The selected genes are listed in **Table 5**. We measured expression of three different reference genes: *HPRT1, GAPDH*, and *RPLP0. HPRT1* was selected as the most suitable for normalization as it was expressed at a relatively similar level to the majority of other transcripts of interest, whereas *GAPDH* and *RPLP0* were very highly expressed. The choice of transcripts tested was informed by previous gene expression studies including transcriptome analysis experiments on samples from The Gambia, Ethiopia and Tanzania and by a genome-wide association study from The Gambia (Holland et al., 2010; Natividad et al., 2010; Burton et al., 2011b,c, 2015; Roberts et al., 2015).

### Statistical analysis

Data were managed in Access and transferred to STATA v14 for analysis. The ophthalmologist's grading of the digital photographs was used for analysis. The ddPCR results were initially exported to R for analysis; the critical cut-off value for designating a positive result for each target was 0.2 plasmid copies/μl of eluted DNA, 0.2 *omcB* copies/μl and >0.3 *RPP30* copies/μl. Samples were designated *C. trachomatis* positive if plasmid and human *RPP30* were detected.

The associations between each of the clinical signs (follicular inflammation, papillary inflammation and scarring) and *C. trachomatis* infection were assessed using univariable logistic regression with infection as the outcome variable. The associations between scarring and each of the other clinical signs, infection, sex and age were investigated with logistic regression, using presence of scarring as the outcome variable. Initially, univariable logistic regression was performed using each exposure in turn, and then a multivariable analysis was performed in order to provide an unbiased estimate of the association between exposure and outcome adjusting for all clinical features.

The gene expression data were normalized relative to the expression of *HPRT1* in the same sample, to adjust for variable concentrations of cDNA. This was done by the ΔC_T_ method. Distributions of ΔC_T_ values were plotted to assess them for normality. Fold change differences in gene expression between different phenotypic groups (Infection, follicular inflammation, papillary inflammation and scarring), vs. individuals without those clinical signs or infection, were calculated using the ΔΔC_T_ method. Linear regression was used to compare the two groups in each comparison, with ΔC_T_ values as the dependent variable and each phenotype group in turn as the independent variable, adjusting for age and sex. To take account of multiple comparisons we used a false discovery rate (FDR) of 5% (Benjamini and Hochberg, 1995). The linear regression analyses were repeated, but rather than taking the four phenotypic groups individually, they were all included as exposures in a multivariable model with age and sex, providing estimates of the fold-changes associated with each phenotype.

A heatmap of ΔC_T_ values for each gene of each participant was produced, to indicate visually whether the expression of individual genes was consistently of higher or lower expression in groups of individuals with clinically active Trachoma (TF and/or TI) and/or infection. A principal component analysis (PCA) was performed on the gene expression data and clinical disease and infection status were overlaid on plots in order to visually indicate any associations between the first two components and infection, active trachoma and scarring. These associations were tested formally using a logistic regression analysis, with the principal components as the exposure variables. For the heatmap and PCA, only individuals with complete expression data could be included. Therefore, so as not to lose too many individuals, any target with >5% of observations missing was excluded from these analyses, as well as the lasso regression and co-expression analyses described below. Any individuals with missing observations on the remaining targets were excluded from these analyses also.

For each of infection, active trachoma and scarring, a multivariable logistic regression was performed using this subset of gene expression levels as exposure variables, with the aim of retaining those expressions most strongly associated with the outcome of interest, adjusting for all other expression levels. Due to the large number of exposure variables, a penalized logistic regression was performed using the lasso technique (Tibshirani, 1997). The list of genes most strongly associated with each outcome was entered into ConsensusPathDB (http://cpdb.molgen.mpg.de/) gene set over-representation analysis, with all genes tested entered as background. Enriched pathway-based sets with a minimum overlap between pathway and input gene list of two and a *p* < 0.05 were identified.

A network graph based on the specimen-to-specimen Pearson correlation was generated using miru (https://kajeka.com/miru). The overall expression correlation matrix and graph were constructed from the raw cycle threshold expression values. The filtered dataset including only individuals and genes with complete expression data was used. Pearson correlation coefficients (r) ≥0.85 were retained and used as cut-offs in network construction. Nodes in the graph are individual mRNA transcripts linked by an edge if r was ≥0.85. The graph was then clustered using a Markov Clustering algorithm with an inflation value of 2.2. The partitioned clusters of expression contain sets of transcripts that exhibit a very high degree of co-expression across the sample set. The co-expression clusters or modules were then investigated for enrichment at the pathway level using ConsensusPathDB as described above, using all genes tested as background. A single value for each cluster of each individual was defined using the first principal component and this value was used to investigate differential expression associated with disease and infection phenotypes.

## Results

### Study participants

A total of 506 children between the ages of 6 and 10 years were recruited and examined. Their demographic and clinical characteristics are described in Table 1. There were almost equal numbers of male and female children, with a mean age of 7.5 years. Most (97.4%) of the children were of the Maasai ethnic group.

**Table 1:** Demographic characteristics, clinical signs and *C. trachomatis* infection status.

**Category**	***n*/506**	**(%)**
Sex (Male)	251	(49.6%)
**ETHNIC GROUP**		
Maasai	493	(97.4%)
Chaga	7	(1.4%)
Sonjo	4	(0.8%)
Pare	2	(0.4%)
Age in years (mean and range)	7.5	(6–10)
**SIMPLIFIED WHO GRADING**		
TF	170	(33.6%)
TI	64	(12.7%)
TS	144	(28.5%)
**DETAILED “FPC” WHO GRADING**		
**Follicles**		
F0	201	(39.7%)
F1	135	(26.7%)
F2	81	(16.0%)
F3	89	(17.6%)
**Papillae**		
P0	180	(35.6%)
P1	166	(32.8%)
P2	96	(19.0%)
P3	64	(12.7%)
**Scarring**		
C0	362	(71.5%)
C1	120	(23.7%)
C2	21	(4.2%)
C3	3	(0.6%)
**DETAILED TARSAL CONJUNCTIVA SCARRING GRADING SYSTEM**		
S0	362	(71.5%)
S1a	71	(14.0%)
S1b	28	(5.5%)
S1c	26	(5.1%)
S2	19	(3.8%)
S3	0	–
***C. trachomatis*** **PLASMID DETECTED**		
No	428	(84.6%)
Yes	78	(15.4%)

*The clinical signs were determined by grading of photographs*.

### Active trachoma

*Trachomatous inflammation-Follicular* (TF, F2/F3) was present in 170 (33.6%) children (Table 1). However, a further 135 (26.7%) had evidence of mild follicular conjunctivitis (F1). Significant conjunctival papillary inflammation (TP, P2/P3) was observed in 160 (31.7%) children, of which 64 (12.7%) had intense papillary inflammation (TI, P3).

### *C. trachomatis* infection and active trachoma

*C. trachomatis* plasmid was detected by ddPCR in 78 (15.4%) individuals. There was a strong association between the presence of TF and the detection of *C. trachomatis* plasmid; 62/78 (79.5%) of *C. trachomatis* positive individuals had TF (Table 2). Of the 16 infected children who did not have TF, 11 (68.8%) had signs of mild follicular conjunctivitis (F1). However, only 62/170 (36.5%) individuals with TF had detectable infection. There was a similar strong association between the presence of TP and chlamydial infection (Table 2).

**Table 2:** *C. trachomatis* plasmid detection and clinical signs; both the simplified and detailed WHO “FPC” grading system.

	***C. trachomatis*** **plasmid**				**OR**	**(95%CI)**	***p*-value**	**Geometric mean**	
	**No**	**(%)**	**Yes**	**(%)**				**Plasmid**	***omcB***
**FOLLICULAR INFLAMMATION**									
No TF	320	(95.2%)	16	(4.8%)	1	–	–	2.99	0.86
TF	108	(63.5)	62	(36.5%)	11.48	(6.4–20.7)	5.95E-16	16.27	4.26
**FPC FOLLICLE SCORE**									
F0	196	(97.5%)	5	(2.5%)	1	–	–	3.41	1.04
F1	124	(91.9%)	11	(8.2%)	3.48	(1.2–10.2)	0.024	2.82	0.81
F2	65	(80.3%)	16	(19.8%)	9.65	(3.4–27.4)	2.03E-05	20.05	4.17
F3	43	(48.3%)	46	(51.7%)	41.93	(15.7–111)	7.97E-14	15.13	4.29
**PAPILLARY INFLAMMATION**									
No TP	323	(93.4%)	23	(6.7%)	1	–	–	4.11	1.23
TP (P2/P3)	105	(65.6%)	55	(34.4%)	7.36	(4.3–12.5)	2.45E-13	17.67	4.64
**FPC PAPILLARY SCORE**									
P0	173	(96.1%)	7	(3.9%)	1	–	–	2.53	0.72
P1	150	(90.4%)	16	(9.6%)	2.63	(1.1–6.6)	0.038	5.09	1.41
P2	73	(76%)	23	(23.9%)	7.79	(3.2–18.9)	6.07E-06	13.47	2.65
P3	32	(50%)	32	(50.0%)	24.71	(10.0–60.8)	2.95E-12	21.47	6.99
**SCARRING**									
No	318	(87.9%)	44	(12.2%)	1	–	–	9.67	2.59
Yes	110	(76.4)	34	(23.6%)	2.23	(1.4–3.7)	0.002	14.38	4.35
**FPC SCARRING SCORE**									
C0	318	(87.9%)	44	(12.2%)	1	–	–	9.67	2.59
C1	89	(74.2%)	31	(25.8%)	2.52	(1.5–4.2)	0.0005	12.33	4.08
C2	18	(85.7%)	3	(14.3%)	1.20	(0.3–4.3)	0.77	70.24	7.85
C3	3	(100%)	0	–				–	–
**DETAILED SCARRING SCORE**									
S0	318	(87.9%)	44	(12.2%)	1	–	–	9.67	2.59
S1a	56	(78.9%)	15	(21.1%)	1.94	(1.0–3.7)	0.047	7.98	3.44
S1b	21	(75.0%)	7	(25.0%)	2.41	(1.0–6.0)	0.059	15.24	4.26
S1c	17	(65.4%)	9	(34.6%)	3.83	(1.6–9.1)	0.0024	21.61	5.32
S2	16	(84.2%)	3	(15.8%)	1.36	(0.4–4.8)	0.64	70.24	7.85
S3	0	–	0	–				–	–

*P-values were calculated by Pearson χ2. TP is equivalent to P2 or P3 of the FPC papillary score*.

**Figure 1:**
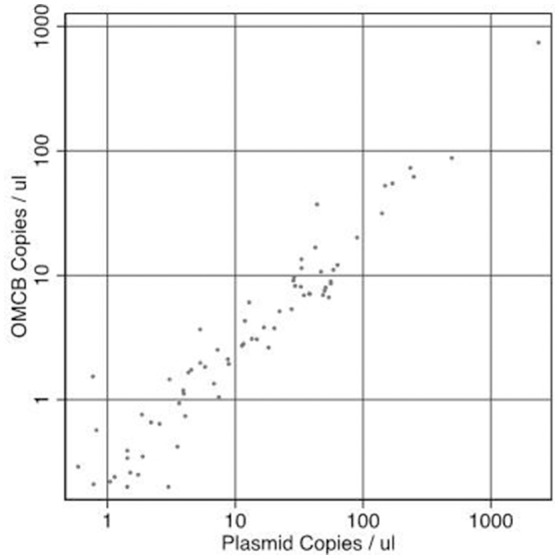
The relationship between paired measures of copies of *Chlamydia trachomatis* plasmid/μl vs. *omcB*/μl in the same sample. Correlation *R*-squared = 0.989.

Infection load and the concentration of *RPP30* endogenous control DNA were quantified. The concentration of plasmid DNA ranged from 0.22 to 3023 copies/μl, *omcB* ranged from 0.2 to 742 copies/μl and *RPP30* ranged from 0.32 to 1,081 copies/μl of eluted DNA. All samples had detectable *RPP30*. There was a marked positive trend in both the proportion infected and the load of infection with increasing *F*-Score and increasing *P*-Score (Table 2). There was a consistent ratio of plasmid to *omcB* copies/μl: geometric mean ratio of 4.57 to 1 (95% CI 3.91–5.34) and a median of 4.79 to 1 (95%CI 4.01–5.21). The correlation between plasmid and *omcB* copies/μl is illustrated in Figure 1. The ratio did not vary with either the follicular or papillary clinical severity scores (data not shown).

### Conjunctival scarring

Conjunctival scarring was relatively frequent, being found in 144 (28.5%) individuals; this was mostly mild scarring (Table 1). There was a significant association between the detection of *C. trachomatis* and scarring [*p* = 0.002, *OR* = 2.23 (95%CI 1.4–3.7)]. The load of infection also increased with scarring severity (Table 2). There were univariate associations between conjunctival scarring and TF, TP (P2/P3), *C. trachomatis* detection and female sex (Table 3). However, in a multivariable model only TP, female sex and age were independently associated with scarring.

**Table 3:** Univariable and multivariable associations between conjunctival scarring and other clinical features, *C. trachomatis* infection, sex and age.

**Variable**	**OR**	**95% CI**	***p*-value**
**UNIVARIABLE ANALYSIS**			
TF	2.28	(1.53–3.40)	5.20E-05
TP (P2/P3)	2.98	(1.99–4.47)	1.22E-07
Plasmid	2.23	(1.36–3.67)	0.002
Sex (Female)	1.76	(1.19–2.61)	0.005
Age	1.06	(0.97–1.17)	0.217
**MULTIVARIABLE LOGISTIC REGRESSION**			
TF	1.39	(0.79–2.44)	0.251
TP (P2/P3)	2.60	(1.52–4.44)	4.83E-04
Plasmid	1.33	(0.75–2.37)	0.326
Sex (Female)	1.63	(1.08–2.45)	0.019
Age	1.16	(1.05–1.29)	0.005

There was strong evidence (*p* = 0.0047) that even at this young age females are proportionately more likely to have signs of established scarring than males (Table 4). Similarly, there was some evidence (*p* = 0.048) that females had slightly increased odds of TP. No evidence was found of an association between sex and the odds of TF or chlamydial infection (*p* = 0.12 and 0.25, respectively) (Table 4).

**Table 4:** The relationship between sex and (i) clinical signs, (ii) *Chlamydia trachomatis* infection and (iii) infection load.

	**Male**	**(%)**	**Female**	**(%)**	**OR**	**(95% CI)**	***p*-value**
**FOLLICULAR INFLAMMATION**							
No TF	175	(69.7%)	161	(63.1%)	1	–	–
TF	76	(30.3%)	94	(36.9%)	1.34	(0.9–1.9)	0.12
**PAPILLARY INFLAMMATION**							
No TP	182	(72.5%)	164	(64.3%)	1	–	–
TP (P2/P3)	69	(27.5%)	91	(35.7%)	1.46	(1.0–2.1)	0.0479
**SCARRING**							
No TS	194	(77.3%)	168	(65.9%)	1	–	–
TS	57	(22.7%)	87	(34.1%)	1.76	(1.2–2.6)	0.0047
**INFECTION**							
No	217	(86.5%)	211	(82.6%)	1	–	–
Plasmid	34	(13.6%)	44	(17.2%)	1.33	(0.8–2.2)	0.25
**GEOMETRIC MEAN AMONG THOSE WITH INFECTION**							
Plasmid	9.45	(4.6–19.3)	13.37	(7.5–23.7)			0.44^α^
*omcB*	2.93	(1.5–5.8)	3.50	(2.1–5.9)			0.67^α^

α *Differences between chlamydial load geometric means in males and females were calculated using a t-test*.

### Conjunctival gene expression

The expression of 91 target genes was measured relative to that of *HPRT1*. Out of the 506 participants, samples from 12 individuals failed the qPCR for all target genes, leaving 494 individuals for gene expression analysis. The number of participant samples with detectable expression for each target is shown in Supplementary Table 1 (N). Multivariable linear regression analysis was performed using data for all 91 genes from 494 individuals.

Four sets of comparisons were performed: (1) infected v non-infected, (2) TF v no TF, (3) TP v no TP, and (4) TS v no TS. The relative Fold Change (FC) between these paired groups, calculated by the ΔΔC_T_ method, are presented in Supplementary Table 1 along with the *p*-values for the linear regression (adjusted for age and sex) for each comparison.

As clinical signs and infection may be highly correlated in their association with the expression of specific targets we tested for independent associations with each target's expression using multivariable linear regression models, Table 5. More genes were significantly differentially expressed in individuals with *C. trachomatis* infection (55/91) relative to individuals with TP (33/91), TF (11/91), and TS (17/91) in these multivariable models (*P* < 0.05). *C. trachomatis* Infection was associated with increased expression of multiple cytokines and chemokines (*CCL2, CXCL13, CCL18, CSF2, FOXP3, IFNG, IDO1, IL1B, IL6, IL8, IL10, IL12B, IL17A, IL19, IL21, IL22, IL23A*), cell cycle components (*CDC25C, TTK, TYMS*), matrix modifiers (MMP7, *MMP9, MMP12, TGFB1*), NK cell markers (*CD247, NCR1, NCAM1*) and intracellular signaling molecules/regulators (*CD274, IKZF1, RHOH, SAMSN1, SERPINB3, SERPINB4, STAT1, STAT4, SOCS1, TBX21*), Table 5. Infection was associated with particularly marked increases in expression of *IFNG* (FC: 7.12, *p*-value: 1.32E-42) and *IL22* (FC: 6.49, *p*-value: 1.6E-23). There was reduced expression of several factors including mucins (*MUC1, MUC4, MUC5AC, MUC7*) and *SPARCL1*.

**Table 5:** Multivariable linear regression models for conjunctival gene expression in the presence of clinical signs, *C. trachomatis* plasmid, female sex and age.

**Target**	**TF**		**TP**		**TS**		**Plasmid**		**Sex (female)^α^**		**Age^β^**	
	**FC**	***p*-value**	**FC**	***p*-value**	**FC**	***p*-value**	**FC**	***p*-value**	**FC**	***p*-value**	**FC**	***p*-value**
**ANTIMICROBIAL PEPTIDES**												
Defensin, beta 4B,defensin, beta 4A (*DEFB4A*)	0.77	0.0919	**2.08**	**2.37E-06**	1.12	0.3844	1.28	0.1459	**1.37**	**0.0044**	**0.92**	**0.0022**
Psoriasin-1 (*S100A7*)	1.40	0.1605	**2.86**	**8.94E-06**	1.25	0.2520	1.77	0.0279	1.36	0.0672	**0.88**	**0.0039**
**CELL CYCLE**												
CD53 molecule (*CD53*)	0.98	0.7932	**1.19**	**0.0033**	1.08	0.1263	1.15	0.0340	1.03	0.4334	0.98	0.1419
M-phase inducer phosphatase 3 (*CDC25C*)	1.17	0.0490	1.08	0.3434	1.04	0.5710	**1.36**	**0.0005**	1.02	0.7103	0.99	0.4182
Cyclin-dependent kinase 13 (*CDK13*)	1.00	0.9318	**0.88**	**0.0037**	1.02	0.6393	0.92	0.0908	1.00	0.8874	1.01	0.1454
Catenin (cadherin-associated protein), delta 2 (*CTNND2*)	1.08	0.7376	0.81	0.3716	1.02	0.9196	0.79	0.3411	0.90	0.5413	1.06	0.1744
Sun Domain Family, Member 6 (*NSUN6*)	0.94	0.0874	0.98	0.5339	1.04	0.2220	1.01	0.8099	0.95	0.0359	1.01	0.1160
Phytanoyl-coa 2-hydroxylase (*PHYH*)	0.92	0.0678	0.95	0.2639	1.01	0.8297	**0.85**	**0.0010**	0.97	0.4291	1.02	0.0196
Tumor protein p53 (*TP53*)	1.01	0.7419	**0.85**	**0.0001**	1.03	0.3965	1.01	0.8485	0.97	0.3148	1.01	0.0460
TTK protein kinase (*TTK*)	1.16	0.0209	1.11	0.0933	1.10	0.0719	**1.31**	**0.0002**	0.98	0.7333	0.99	0.4067
Thymidylate synthetase (*TYMS*)	1.11	0.0590	**1.16**	**0.0050**	1.00	0.9654	**1.62**	**6.38E-15**	1.05	0.2448	0.99	0.1936
**CYTOKINES/CHEMOKINES**												
Chemokine ligand 18 (*CCL18*)	1.00	0.9903	**3.03**	**6.14E-10**	0.94	0.6534	**1.99**	**0.0004**	0.97	0.8422	**0.89**	**0.0006**
Chemokine ligand 2 (*CCL2*)	0.95	0.7092	**1.63**	**0.0008**	**1.39**	**0.0060**	**3.36**	**1.45E-13**	1.13	0.2284	**0.87**	**1.06E-07**
Chemokine ligand 20 (*CCL20*)	**1.45**	**0.0005**	1.04	0.7406	**1.33**	**0.0011**	1.23	0.0735	0.97	0.7140	**0.95**	**0.0035**
Chemokine receptor 6 (*CCR6*)	**1.53**	**0.0011**	0.83	0.1466	1.11	0.3398	1.10	0.4912	1.12	0.2129	0.96	0.1173
Colony stimulating factor 2 (*CSF2*)	1.19	0.0798	1.05	0.6564	1.13	0.1416	**2.45**	**2.83E-15**	1.14	0.0690	0.99	0.5702
Colony stimulating factor 3 (*CSF3*)	0.97	0.8494	1.40	0.0206	**1.52**	**0.0005**	0.85	0.3008	0.99	0.8901	**0.91**	**0.0008**
Chemokine ligand 13 (*CXCL13*)	1.41	0.0321	**2.01**	**1.22E-05**	1.14	0.3140	**2.11**	**2.29E-05**	1.23	0.0733	**0.87**	**3.46E-06**
Chemokine ligand 5 (*CXCL5*)	1.11	0.4860	**1.82**	**0.0001**	**1.60**	**0.0001**	0.82	0.2278	0.88	0.2371	**0.88**	**6.49E-06**
Forkhead box P3 (*FOXP3*)	1.02	0.7750	1.02	0.7818	0.91	0.0840	**1.25**	**0.0014**	0.95	0.2135	1.00	0.9477
Indoleamine 2,3-dioxygenase 1 (*IDO1*)	1.17	0.1491	1.28	0.0187	**1.24**	**0.0139**	**1.73**	**3.53E-06**	**1.34**	**0.0001**	**0.89**	**1.05E-08**
Interferon gamma(*IFNG*)	**1.36**	**0.0109**	1.09	0.4791	1.12	0.2396	**7.12**	**1.32E-42**	**1.29**	**0.0025**	**0.91**	**2.97E-05**
Interleukin 10 (*IL10*)	1.13	0.2184	**1.49**	**3.48E-05**	**1.22**	**0.0141**	**1.78**	**7.86E-08**	1.09	0.2106	**0.94**	**0.0004**
Interleukin 12 beta (*IL12B*)	1.15	0.2315	1.19	0.1340	1.07	0.4853	**3.42**	**3.90E-21**	**1.23**	**0.0106**	0.97	0.2047
Interleukin 13 (*IL13*)	1.63	0.1091	0.66	0.1804	0.80	0.2701	1.38	0.2585	1.03	0.8545	1.07	0.1290
Interleukin 17A (*IL17A*)	1.16	0.2561	**1.65**	**0.0001**	1.19	0.0928	**2.34**	**1.85E-09**	1.18	0.0688	**0.90**	**3.56E-06**
Interleukin 19 (*IL19*)	0.96	0.7566	**2.63**	**5.69E-11**	1.16	0.2150	**2.11**	**3.75E-06**	**1.43**	**0.0006**	**0.88**	**6.69E-07**
Interleukin 1 beta(*IL1B*)	0.97	0.8261	**1.72**	**5.46E-06**	**1.36**	**0.0021**	**1.55**	**0.0009**	1.04	0.6251	**0.92**	**0.0002**
Interleukin 21 (*IL21*)	**1.68**	**0.0003**	1.36	0.0290	**1.34**	**0.0132**	**3.10**	**1.69E-12**	1.16	0.1432	**0.90**	**0.0001**
Interleukin 22 (*IL22*)	1.14	0.4452	1.26	0.1548	1.31	0.0511	**6.49**	**1.60E-23**	1.22	0.0973	0.93	0.0302
Interleukin 23A (*IL23A*)	0.98	0.8604	**1.70**	**3.81E-08**	1.10	0.2287	**1.87**	**3.62E-09**	0.99	0.8875	**0.93**	**3.13E-05**
Interleukin 33 (*IL33*)	0.96	0.6351	0.92	0.3465	**1.28**	**0.0009**	1.27	0.0164	0.96	0.5468	1.03	0.0472
Interleukin 6 (*IL6*)	0.92	0.5490	1.27	0.0721	1.27	0.0303	**1.80**	**0.0001**	0.92	0.3965	**0.94**	**0.0057**
Interleukin 8 (*IL8*)	0.87	0.1443	**1.43**	**0.0001**	**1.24**	**0.0041**	**1.32**	**0.0063**	1.00	0.9975	0.97	0.0751
Prostaglandin-endoperoxide synthase 2 (*PTGS2*)	0.90	0.3527	**1.45**	**0.0009**	**1.26**	**0.0116**	1.18	0.1781	0.98	0.7886	0.97	0.0978
Tumor necrosis factor (*TNF*)	**1.39**	**0.0040**	0.93	0.4934	1.16	0.1187	1.30	0.0383	1.15	0.0965	1.00	0.8808
**EMT MARKERS**												
Alpha smooth muscle actin (*ACTA2*)	1.10	0.1559	**0.83**	**0.0039**	1.14	0.0144	**1.29**	**0.0004**	1.04	0.3549	0.99	0.4144
Epithelial cadherin (*CDH1*)	0.94	0.2482	0.93	0.1746	1.05	0.2799	**0.78**	**4.22E-05**	0.99	0.7167	1.00	0.9175
Cadherin 1, type 1, E-cadherin (epithelial) (CDH1)	0.95	0.3727	0.92	0.1964	1.04	0.4101	**0.73**	**3.06E-06**	0.99	0.8120	1.01	0.5096
Neuronal cadherin (*CDH2*)	0.98	0.8576	**0.71**	**0.0066**	1.18	0.1088	1.11	0.4339	1.00	0.9960	1.04	0.0558
S100 calcium binding protein A4 (*S100A4*)	0.88	0.0691	**0.71**	**1.83E-06**	1.15	0.0213	**0.47**	**1.99E-20**	0.90	0.0393	**1.03**	**0.0093**
Vimentin (*VIM*)	1.02	0.6112	1.04	0.4486	1.04	0.2589	**1.51**	**8.85E-15**	1.01	0.7266	1.01	0.3843
**MATRIX MODIFIERS**												
Connective tissue growth factor (*CTGF-1*)	0.98	0.8349	0.81	0.0191	0.98	0.7534	1.20	0.0721	**0.79**	**0.0003**	1.02	0.2769
Connective tissue growth factor (*CTGF-2*)	0.89	0.2310	0.85	0.0721	0.91	0.2245	1.21	0.0675	**0.82**	**0.0036**	1.03	0.0828
Fibroblast growth factor 2 (basic) (*FGF2*)	0.86	0.3151	1.11	0.4830	**1.43**	**0.0032**	**1.48**	**0.0124**	0.87	0.1988	1.01	0.7147
Matrix metallopeptidase 12 (*MMP12*)	1.31	0.0246	**1.48**	**0.0010**	1.26	0.0188	**2.00**	**1.84E-07**	1.11	0.2136	**0.92**	**0.0002**
Matrix metallopeptidase 7 (*MMP7*)	0.94	0.5657	**1.41**	**0.0024**	1.04	0.7042	**0.38**	**1.63E-14**	0.93	0.3375	0.96	0.0488
Matrix metallopeptidase 9 (*MMP9*)	**1.37**	**0.0048**	**1.43**	**0.0012**	1.20	0.0519	**1.77**	**3.45E-06**	0.98	0.8380	0.96	0.0371
Platelet-derived growth factor beta polypeptide (*PDGFB*)	1.00	0.9393	1.08	0.1904	**1.16**	**0.0043**	**1.43**	**1.55E-07**	1.06	0.1638	**0.96**	**0.0002**
SPARC-like 1 (hevin) (*SPARCL1*)	**0.53**	**0.0048**	**0.39**	**3.20E-05**	0.85	0.3842	**0.46**	**0.0020**	0.75	0.0794	**1.20**	**1.29E-05**
Transforming growth factor, beta 1 (*TGFβ1*)	1.02	0.6999	0.99	0.7693	1.08	0.0491	**1.23**	**0.0001**	0.99	0.6965	0.98	0.0305
Transforming growth factor, beta 2 (*TGFβ2*)	0.97	0.7251	0.96	0.6469	1.08	0.3172	0.91	0.3438	0.92	0.2258	1.01	0.6926
**RESPONSE TO MICROBIOTA**												
Arachidonate 5-lipoxygenase (*ALOX5*)	**0.88**	**0.0111**	0.97	0.4844	1.05	0.2353	**0.67**	**1.78E-13**	0.97	0.4387	1.01	0.2310
B-cell CLL/lymphoma 2 (*BCL2*)	1.00	0.9769	0.94	0.1509	1.09	0.0239	1.13	0.0212	0.97	0.4448	1.00	0.8130
CD40 molecule, TNF receptor superfamily member 5 (*CD40*)	0.98	0.6989	0.98	0.7901	1.06	0.2693	**1.25**	**0.0009**	1.06	0.1592	1.00	0.8334
Dual oxidase 2 (*DUOX2*)	0.92	0.3364	**1.38**	**0.0004**	1.09	0.2734	1.19	0.0852	**1.20**	**0.0047**	**0.94**	**0.0001**
V-rel avian reticuloendotheliosis viral oncogene homolog (*REL*)	0.96	0.3595	1.08	0.0911	1.05	0.1697	1.03	0.5774	1.05	0.1108	1.00	0.6653
Tumor necrosis factor receptor superfamily, member 1A (*TNFRSF1A*)	0.87	0.1556	0.99	0.9463	1.07	0.3766	0.81	0.0448	0.96	0.5130	1.02	0.3991
Tumor necrosis factor receptor superfamily, member 1B (*TNFRSF1B*)	0.89	0.1026	1.18	0.0164	1.10	0.0956	1.02	0.8439	0.96	0.4386	1.00	0.7988
**MUCINS**												
Mucin 1, cell surface associated (*MUC1*)	0.92	0.1555	1.04	0.4586	1.03	0.4947	**0.82**	**0.0013**	1.00	0.9901	1.00	0.8866
Mucin 4, cell surface associated (*MUC4*)	0.93	0.3097	1.15	0.0472	1.06	0.3140	**0.70**	**1.02E-05**	0.96	0.4576	0.98	0.0865
Mucin 5AC, oligomeric mucus/gel-forming (*MUC5AC*)	0.77	0.0561	**0.61**	**0.0002**	1.25	0.0464	**0.56**	**0.0001**	0.97	0.7651	1.04	0.0811
Mucin 7, secreted (*MUC7*)	0.86	0.3401	0.69	0.0217	0.94	0.6225	**0.42**	**1.48E-06**	**0.63**	**0.0001**	**1.10**	**0.0012**
**NK CELL MARKERS**												
*CD247* molecule (*CD247*)	1.12	0.0847	1.02	0.7505	1.00	0.9863	**1.80**	**1.78E-15**	1.06	0.2277	0.98	0.0376
Neural cell adhesion molecule 1 (*NCAM1*)	0.85	0.0590	**0.76**	**0.0007**	1.14	0.0524	**1.79**	**3.62E-10**	0.92	0.1385	1.00	0.9652
Natural cytotoxicity triggering receptor 1 (*NCR1*)	1.11	0.1621	1.00	0.9473	1.13	0.0531	**2.31**	**1.68E-22**	1.07	0.2349	**0.95**	**0.0002**
**PATTERN RECOGNITION RECEPTORS**												
Nucleotide-binding oligomerization domain containing 2 (*NOD2*)	0.88	0.1748	1.21	0.0348	1.13	0.1221	1.08	0.4293	0.90	0.0932	0.99	0.4252
Toll-like receptor 2 (*TLR2*)	1.01	0.8677	1.01	0.9108	**1.26**	**0.0013**	0.84	0.0615	1.02	0.7880	0.98	0.1690
Toll-like receptor 4 (*TLR4*)	0.99	0.9140	1.20	0.0184	**1.20**	**0.0039**	1.02	0.8485	0.94	0.2395	0.97	0.0550
**REGULATORS/SIGNALING PATHWAYS**												
CD274 molecule (*CD274*)	1.01	0.8828	**1.44**	**0.0001**	1.16	0.0507	**2.47**	**1.18E-18**	**1.20**	**0.0046**	**0.95**	**0.0030**
Chromodomain helicase DNA binding protein 8 (*CHD8*)	0.93	0.0593	1.01	0.7501	1.04	0.2423	1.00	0.9029	0.98	0.4950	1.01	0.2923
COMM domain containing 6 (*COMMD6*)	0.99	0.9205	0.96	0.6492	1.00	0.9510	0.89	0.2836	0.99	0.8589	0.99	0.6788
Hematopoietically expressed homeobox (*HHEX*)	**1.34**	**8.54E-06**	0.92	0.2204	1.07	0.1923	1.11	0.1411	1.04	0.3702	1.00	0.7584
IKAROS family zinc finger 1 (Ikaros) (*IKZF1*)	1.11	0.0319	0.99	0.9105	1.04	0.3969	**1.37**	**1.44E-08**	1.00	0.9898	1.01	0.2868
Myeloid differentiation primary response 88 (*MYD88*)	0.93	0.0303	1.05	0.1445	1.07	0.0151	1.00	0.9773	1.01	0.6856	1.00	0.6936
Marginal zone B and B1 cell-specific protein (*MZB1*)	1.16	0.2162	1.24	0.0712	0.95	0.6338	**2.07**	**8.27E-08**	1.21	0.0279	0.98	0.3593
Nuclear factor of kappa light polypeptide gene enhancer in B-cells 1 (*NFKB1*)	0.95	0.1251	1.06	0.1139	1.04	0.1457	**1.13**	**0.0022**	0.99	0.7545	1.00	0.5891
Phosphatidylinositol-3,4,5-trisphosphate-dependent Rac exchange factor 2 (*PREX2v1*)	1.06	0.5998	0.81	0.0744	0.90	0.2736	**1.38**	**0.0115**	0.95	0.5644	1.02	0.4858
Phosphatidylinositol-3,4,5-trisphosphate-dependent Rac exchange factor 2 (*PREX2v2*)	1.07	0.5708	1.08	0.5194	0.84	0.0985	1.05	0.6832	0.86	0.0769	1.03	0.1608
Ras homolog family member H (*RHOH*)	**1.20**	**0.0034**	1.01	0.8137	1.03	0.6026	**1.47**	**4.37E-08**	1.11	0.0183	0.97	0.0227
SAM domain, SH3 domain and nuclear localization signals 1 (*SAMSN1*)	1.03	0.6236	**1.32**	**4.02E-05**	1.04	0.5155	**1.36**	**0.0001**	1.05	0.2859	0.97	0.0229
Serpin peptidase inhibitor B3 (*SERPINB3*)	0.87	0.3173	**1.90**	**6.73E-06**	1.32	0.0175	**2.65**	**8.33E-10**	0.86	0.1311	0.94	0.0185
Serpin peptidase inhibitor clade B member 4, (*SERPINB4*)	0.75	0.2648	**2.85**	**0.0001**	1.25	0.3075	**4.38**	**1.95E-07**	0.97	0.8763	1.02	0.7506
Suppressor of cytokine signaling 1 (*SOCS1*)	1.15	0.0606	1.10	0.2035	1.09	0.1646	**2.06**	**1.08E-17**	1.05	0.3178	**0.94**	**6.95E-06**
Suppressor of cytokine signaling 3 (*SOCS3*)	1.01	0.9205	**1.50**	**4.53E-05**	**1.34**	**0.0004**	1.21	0.0777	1.02	0.7518	**0.93**	**0.0001**
Serglycin (*SRGN*)	0.91	0.2568	**1.28**	**0.0018**	1.12	0.0767	0.97	0.7165	0.98	0.7288	0.99	0.6097
Signal transducer and activator of transcription 1 (*STAT1*)	1.02	0.8024	1.12	0.0565	1.11	0.0391	**1.97**	**1.34E-22**	1.09	0.0392	**0.95**	**9.97E-07**
Signal transducer and activator of transcription 3 (*STAT3*)	0.93	0.1345	1.06	0.1683	**1.14**	**0.0007**	1.00	0.9584	0.96	0.2153	0.98	0.0423
Signal transducer and activator of transcription 4 (*STAT4*)	1.02	0.8085	1.00	0.9741	1.04	0.4556	**1.85**	**5.37E-18**	1.04	0.3692	0.98	0.0535
T-box 21 (*TBX21*)	**1.21**	**0.0044**	1.00	0.9917	1.07	0.2307	**2.18**	**6.56E-25**	1.11	0.0197	**0.97**	**0.0060**
Ubiquitin specific peptidase 6 (Tre-2 oncogene) (*USP6*)	0.70	0.1911	0.87	0.6216	0.72	0.1582	0.97	0.9124	0.65	0.0322	0.99	0.7936

α *FC>1 indicates expression was greater in females than males*.

β *FC calculated with an age unit increase of 1 year*.

*FC = fold change. Using the Benjamini and Hochberg approach for adjusting for multiple comparisons, in order to control the false discovery rate <5% only tests with a p-value below 0.0142 (highlighted in bold) are considered statistically significant*.

Clinical signs of active trachoma were associated with increased expression of multiple cytokines, chemokines, antimicrobial peptides, matrix modifiers, following a similar pattern to infection (Table 5). TP was associated with more substantial increases in a wider range of factors than TF (Table 5): antimicrobial peptides (*DEFB4A, S100A7*), cytokine/chemokines (*CXCL5, CXCL13, CCL18, CCL2, IL1B, IL8, IL10, IL17A, IL19, IL23A, PTGS2*), matrix modifiers (*MMP7, MMP9, MMP12*) and intracellular signaling molecules/regulators (*CD274, SAMSN1, SERPINB3, SERPINB4, SOCS3*). Several cytokines and chemokines (*CCL18, CXCL13, IL19*, and *S100A7*) had a FC >2 in children with TP. Conjunctival scarring was associated with modestly increased expression (>1.2 FC) and borderline significance of several chemokines and cytokines (*CCL2, CCL20, CXCL5, CSF3, IL1B, IL8, IL10, IL21, IL33, FGF2*) in the multivariable linear regression models (Table 5). *SPARCL1* was significantly downregulated in TF and TP but not TS.

**Figure 2:**
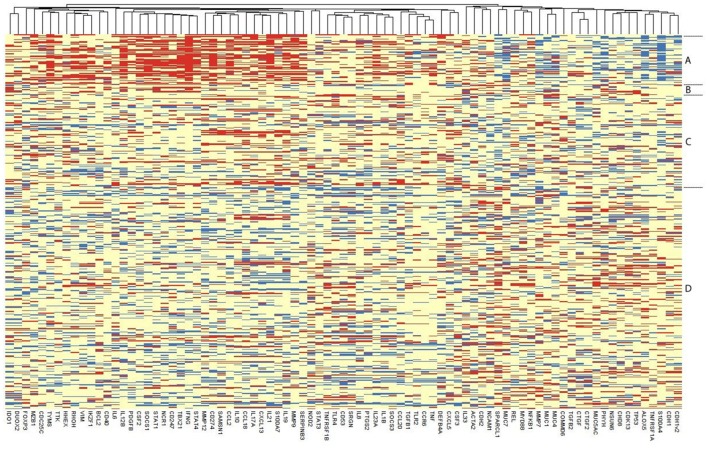
Heatmap of the expression of each of the 83 retained genes in 458 individuals. Each row represents an individual, with individuals grouped as per their disease and infection status and the relevant group indicated on the right: Group **(A)** Infected, Active Trachoma; **(B)** Infected, no Active Trachoma; **(C)** Uninfected, Active Trachoma; **(D)** Uninfected, no Active Trachoma. ΔC_T_ values of genes are represented in the columns, with genes that tend to be expressed together located next to one another. Red means that an individual has a higher expression of that gene, blue, a lower expression, relative to that individual's expression of the endogenous control gene *HPRT1*.

Only 108 individuals had detectable gene expression for all 91 targets, so in order to retain a reasonable number of individuals in the PCA and lasso analyses, which require complete records for all individuals, targets which were missing >5% of observations were excluded. This resulted in the exclusion of eight transcripts (*USP6, IL13, CTNND2, FGF2, SERPINB4, IL22, PREX2v1, PREX2v2*) for which there was no detectable expression in >5% individuals. In individuals with detectable expression of these eight targets, raw C_T_ values were generally very high (C_T_ > 33). These eight targets were excluded from the heatmap, PCA, lasso regression and co-expression network analyses, retaining a complete expression dataset for 83 genes. Among the 494 individuals, 36 were excluded as they had missing data among the 83 included targets, resulting in a final dataset of 458 individuals and 83 genes. Excluding further genes offered minimal gains in sample size. A heatmap of ΔC_T_ values separated by clinical and infection phenotype is presented in Figure 2. Infected individuals with or without active trachoma had a visibly distinct pattern of gene expression, whereas the ΔC_T_ values of normal healthy controls and individuals with active trachoma in the absence of infection appeared more similar overall.

PCA analysis was performed for 458 individuals that had complete gene expression data for 83 targets and active trachoma/infection and scarring status were overlaid on plots, Supplementary Figures 1A,B. Principal component 1 (PC1) was strongly associated with infection and active trachoma; for a one unit increase in PC1 there was an estimated 35% decrease in the odds of infection (95% CI 27–41%, *p* < 0.001) and an estimated 23% decrease in the odds of active trachoma (95% CI 9–28%, *p* < 0.001). PC1 loadings showed that as genes in the chemokines/cytokines and regulator/signaling pathways groups were up-regulated, PC1 became smaller, suggesting that the odds of infection/active trachoma was greater among individuals where these genes are up-regulated. The expression of mucins, *CDH1, SPARCL1*, and *S100A4* showed the reverse relationship, as when these genes were down-regulated the PC1 was smaller. Principal component 2 (PC2) was associated with increased odds of active trachoma alone, with a unit increase in PC2 being associated with an estimated 15% increase in the odds (95% CI 7–24%, *p* < 0.001), however there was no evidence of an association between PC2 and infection. There was evidence of a weaker association between PC1 and scarring, with an estimated 10% decrease in the odds of scarring (95% CI 6–14%, *p* < 0.001) per unit increase in PC1, Supplementary Figure 1B. There was no evidence of an association between scarring and PC2.

Three lasso logistic regressions were performed, first using infection (with or without active disease) as the outcome and the 83 targets, age and sex of 458 individuals as exposures. Then, excluding all infected individuals, active trachoma (uninfected only) was used as the outcome against uninfected individuals without active trachoma and the same 83 targets, age and sex as the exposures of interest. Finally, the regression was performed using scarring as the outcome and the same set of exposures in all 458 individuals. The transcripts most strongly associated with each of the three outcomes, and biological pathways with members that were over-represented in each relative to the background (83 targets) are listed in Table 6. Genes most strongly associated with infection were associated with Th1 cell development and NK cell pathways, whilst genes most associated with active trachoma in the absence of *C. trachomatis* infection were enriched for members of leukocyte transendothelial migration, TNF receptor, MMP activation, collagen formation and collagen fibrils assembly pathways. Genes most strongly associated with the TS phenotype were enriched for members of immunoregulatory interactions between lymphoid and non-lymphoid cells.

**Table 6:** Lasso logistic regression of gene expression by infection, active disease (in only uninfected individuals) and scarring status.

***C. trachomatis* infection**	**Active trachoma (uninfected only)**	**Scarring**
IFNG	HHEX	IL33
IL17A	CDH1v2	CXCL5
NCAM1	MMP9	IDO1
MZB1	MMP7	CCL2
CTGF	IDO1	MUC5AC
NCR1	IL21	IL21
TYMS	IFNG	MMP9
SERPINB3	CXCL5	TLR2
IKZF1	RHOH	PDGFB
MUC7	TNFRSF1B	CD40
CD53	S100A4	CDH2
IL21	SPARCL1	IL10
IL12B	VIM	SERPINB3
SOCS1	MYD88	NCR1
TBX21	TNFRSF1A	TTK
S100A7	NCAM1	BCL2
IL10	FOXP3	SOCS3
S100A4		FOXP3
MMP7		CCL18
CSF3		CTGF2
ALOX5		COMMD6
PDGFB		SAMSN1
ACTA2		CD247
CDC25C		
CDH2		
FOXP3		
STAT4		
IL6		
**OVER-REPRESENTED PATHWAYS**		
IL-12 and STAT4 dependent signaling pathway in Th1 development [15:3/3]^*^	Inflammatory Response Pathway [32:3/4]	Immunoregulatory interactions between a Lymphoid and a non-Lymphoid cell [211:3/4]
NO2-dependent IL-12 pathway in NK cells [9:3/3]	Activation of Matrix Metalloproteinases [31:2/2]	Signaling events mediated by PTP1B [53:3/4]
	Assembly of collagen fibrils and other multimeric structures [49:2/2]	
	Leukocyte transendothelial migration [116:2/2]	
	Collagen formation [94:2/2]	
	TNFs bind their physiological receptors [30:2/2]	

* *[x:y/z] x = total number of genes in pathway, y = number of pathway genes present in input list, z = number of pathway genes present in background*.

*For each outcome, the genes whose expression was most strongly associated are listed. Biological pathways with members that are over-represented (P ≤ 0.05) in each gene list relative to the background (all genes tested) are shown for each phenotype, in descending order*.

Networks of co-expression, independent of differential expression, were explored in the complete expression data of 83 transcripts and 458 individuals using Miru. The undirected graph contained 51 nodes connected by 121 edges. Markov clustering partitioned the network into 5 separate clusters of co-expressed genes that accounted for 38/51 (74.5%) of the transcripts in the original network. Cluster 1 contained 11 co-expressed transcripts, cluster 2 contained 10 transcripts, there were 6 transcripts in each of cluster 3 and 4, and 5 transcripts in cluster 5 (Supplementary Table 2A), the remaining nodes were not connected in the graph. The most connected gene transcript or hub in each module was MyD88/REL (cluster 1), IKZF1/VIM (cluster 2), STAT1 (cluster 3), SRGN (cluster 4), and IL10/IL17A/IL21 (cluster 5).

Pathway enrichment analysis was performed for each cluster relative to the background of 83 transcripts (Supplementary Table 2B). ΔC_T_ values for the transcripts in each cluster were collapsed into a single value for each of the 458 individuals, represented by the first principal component. Multivariable linear regression was then performed for each cluster, using the first principal component as the outcome variable and TF, TP, TS, infection, age, and sex as independent variables (Supplementary Table 2C). Cluster 1 was enriched for cell cycle and apoptosis pathways, however the combined expression value was not differentially expressed in any condition. Cluster 2 was significantly associated with infection and TF and was marginally enriched for the retinoblastoma pathway. Cluster 3 was highly enriched for Th1/2 cell differentiation, IFNγ, IL-12, CD8, and NK cell signaling pathways and was strongly associated with infection and age and marginally associated with sex, but interestingly not with TP or TF. Conversely, cluster 4 was enriched for TNF, inflammasome, TLR and NFkB signaling pathways and was associated with TP, TS, and age but not infection. Cluster 5 was enriched for the allograft rejection pathway and was associated with infection, TP, TS and age.

## Discussion

### Clinical disease and *C. trachomatis* infection

In this study, conducted in communities prior to azithromycin MDA, there was a relatively high TF (34%) and moderate *C. trachomatis* (15.4%) prevalence in 6–10 year olds. There was a strong relationship between infection and clinical signs. The large majority (79.5%) of individuals with *C. trachomatis* detected by ddPCR had TF (F2/F3). Moreover, most individuals (68.8%) with detectable chlamydial infection but without TF had a mild follicular conjunctivitis (F1). There were few cases of infection in the absence of clinical signs. This is consistent with earlier studies which found a stronger correlation as the underlying prevalence of infection increased (Ramadhani et al., 2016b). However, in common with many other studies reporting the relationship between disease and infection, a minority (36%) of individuals with TF had detectable infection, probably due to the shorter duration of infection episodes relative to disease (Grassly et al., 2008; Burton et al., 2011a,c; Lee et al., 2014). With increasing severity of both TF and TP scores, there was an increasing proportion with infection and increasing loads of infection. Consistent with earlier studies, increasing load appeared to be more closely related to increasing TP rather than TF (Burton et al., 2003; Solomon et al., 2003; Michel et al., 2011; Derrick et al., 2016a).

In the present cross-sectional study conjunctival scarring was associated with TP, female sex and increasing age but not *C. trachomatis* infection, which is consistent with several earlier cohort studies (Dawson et al., 1990; West et al., 2001; Wolle et al., 2009b; Burton et al., 2015; Hu et al., 2016). However, longitudinal data supporting an association between TF or *C. trachomatis* infection and progressive scarring are limited (Ramadhani et al., 2016a). We found females had more TP and TS than males, whereas *C. trachomatis* infection and TF prevalence were not significantly different between the sexes (Table 4). This might suggest that there are additional determinants of TP and TS development beyond the initial *C. trachomatis* infection. It is generally believed that the difference in the proportion of males and females developing scarring sequelae of trachoma is attributable to a greater life-time exposure to repeated *C. trachomatis* infection among females (Taylor et al., 2014). While that may well be the case, the data from this study also suggest that even at a relatively young age, females appear more susceptible to developing TP and TS than males, despite comparable levels of *C. trachomatis* infection. This finding is consistent with the observation that in general, females generate stronger immune responses than males, making them more susceptible to diseases resulting from immune-mediated pathology (Klein and Flanagan, 2016).

Among infected individuals, the *C. trachomatis* plasmid:*omcB* ratio (4.57:1) was very similar to the ratio reported from a range of ocular and genital *C. trachomatis* isolates (mean 4.0:1) and from a population-based trachoma study in Guinea Bissau (5.3:1) (Pickett et al., 2005; Last et al., 2014). In common with the study from Guinea Bissau, we did not find evidence of variation in the plasmid copy number with increasing disease severity scores.

### Host gene expression

*IFNG* was strongly upregulated in association with *C. trachomatis* infection, consistent with previous findings in trachoma endemic populations (Burton et al., 2004; Holland et al., 2006; Natividad et al., 2010). Several NK cell markers were also upregulated in association with *C. trachomatis* infection: *NCR1, CD56 (NCAM1)*, and *CD247. IFN*γ, *NCAM1*, and *NCR1* were among the genes most strongly associated with increased odds of *C. trachomatis* infection, and Th1 and NK cell pathways were enriched in this gene set (Table 6). A strong *IFNG* response and transcriptional signatures associated with NK cell activation and cytotoxicity were closely associated with *C. trachomatis* infection and active trachoma in a West African population (Natividad et al., 2010), and infiltrates suggestive of NK cells were identified in scarred conjunctival biopsy tissue from individuals with trachomatous trichiasis from Tanzania (Hu et al., 2016). In peripheral blood from adults with trichiasis and controls that were stimulated *ex-vivo* with *C. trachomatis* antigens, NK cells and to a lesser extent CD8+ T cells were the major sources of IFNγ with <50% of the IFNγ produced by CD4+ T cells (Gall et al., 2011). In a non-human primate live-attenuated ocular chlamydial vaccine study, depletion of CD8+ cells abrogated protective immunity, however the contribution of CD4+ and NK cells was not investigated (Olivares-Zavaleta et al., 2014). IFNγ is a crucial component of the Th1 response to restrict *C. trachomatis* survival via tryptophan depletion, macrophage activation and promoting leukocyte adhesion, migration and activation (Beatty et al., 1994; Mosser and Edwards, 2008; Redgrove and McLaughlin, 2014). Interestingly, whilst *IFNG* was strongly upregulated in individuals with *C. trachomatis* infection, there was much less upregulation in individuals with disease but not infection, suggesting that the *IFNG* response is quickly down-regulated once *C. trachomatis* has been cleared. Supporting this observation, cluster 3, which was enriched for Th1, NK, IL-12, and IFNγ pathways, was strongly associated with infection but not with TF or TP. Cluster 3 expression was also marginally associated with female sex, and *IFNG, IDO1, IL12B*, and *IL19* were each upregulated in females relative to males (Table 5). Both the number of activated CD4+ cells and their relative expression of pro-inflammatory genes including *IFNG* have previously been shown to be higher in females than males (Hewagama et al., 2009; Sankaran-Walters et al., 2013). Our data suggest that increased expression of *IFNG* and related pathways in females may contribute to the observed differences in tissue responsiveness (TP and TS) between the sexes in the context of equal infection exposure. In support of this hypothesis, a longitudinal study conducted in The Gambia that visited participants every 2 weeks found that the likelihood of developing infection or active disease and the duration of infection were independent of sex, however males had a reduced duration of active disease relative to females (Grassly et al., 2008). Whilst Th1 responses and *IFNG* are essential for controlling chlamydial infection, excessive responses are thought to cause collateral tissue damage and contribute to chlamydial pathology (Van Voorhis et al., 1997; Rank et al., 2000). It is therefore possible that a female predisposition to overproduce Th1 responses and *IFNG* may tip the balance from protection toward pathological tissue damage. However, further research is required to clarify this hypothesis and a direct causal relationship cannot be determined from this cross-sectional analysis.

The cytokine genes *IL1*β, *IL6, IL17, IL21, IL22, IL23*, and *TGF*β*1* were strongly expressed in individuals with *C. trachomatis* infection. Th17 cells are generated in the presence of TGFβ1, IL-1β, IL-6, IL-21, and IL-23 and classically produce IL-17, IL-22, and IL-21 (Manel et al., 2008; Zielinski et al., 2012). *IL21* expression was also upregulated in individuals with TF and TS. Individuals with TP significantly upregulated *IL17* and *IL23* expression and *IL21* at marginal significance. The combined expression of cluster 5, containing *IL10, IL17A, IL21, CXCL13*, and *MMP12*, was significantly associated with *C. trachomatis* infection, TF, TP, and TS. Th17 cells have important roles in mucosal immunity against bacteria and fungi, however they are also associated with the inflammatory pathology of diseases such as Crohn's disease, psoriasis and rheumatoid arthritis (Tesmer et al., 2008). Epithelial cells stimulated by IL-17 and IL-22 upregulate chemokines, cytokines, antimicrobial peptides and growth factors such as IL-6, IL-8, S100A7, and GM-CSF (CSF2) (Wolk et al., 2006; Eyerich et al., 2010); these transcripts were upregulated in individuals with *C. trachomatis* infection (*S100A7* at marginal significance). *IL8* and *S100A7* were also upregulated in individuals with TP. IL-8 and S100A7, amongst other factors induced by Th17 cells, are chemotactic for neutrophils, an influx of which is associated with *C. trachomatis* infection and contributes to pathology in animal models of urogenital and ocular infection (Frazer et al., 2011; Lacy et al., 2011). IL-22, which was highly upregulated with infection, is also important in the maintenance of epithelial health and barrier function (Radaeva et al., 2004; Rutz et al., 2013). The relative contributions of *IL-17* and associated responses to protection from *C. trachomatis* infection and maintenance of barrier function vs. pathological tissue damage remain unclear. In murine models of genital *C. trachomatis* infection higher levels of IL-17/Th17 cells have been associated with greater pathology (Lu et al., 2012; Vicetti Miguel et al., 2016), whereas in IL-23 knock-out mice which had greatly reduced levels of IL-17 and IL-22 there was no difference in infection burden or oviduct pathology compared to wild type mice (Frazer et al., 2013). Longitudinal investigation of these factors is expected to further our understanding of the role of Th17 cell associated cytokines in human chlamydial pathogenesis.

*STAT1* and *STAT4*, which promote Th1 cell development (Nishikomori et al., 2002; Mikhak et al., 2006; Zhu et al., 2010), were upregulated in response to *C. trachomatis* infection. *STAT1* and *STAT4* have previously been shown to be associated with active trachoma and ocular *C. trachomatis* infection, and *STAT1* was upregulated in cervical epithelial cells infected with *C. trachomatis in vitro* which was found to inhibit chlamydial growth (Lad et al., 2005; Natividad et al., 2010). *STAT3*, which is required for Th17 development (Zhu et al., 2010; Yang et al., 2011), was not differentially regulated, contrasting the upregulation of Th17 cytokines. *SOCS1, IL10*, and *FOXP3* were upregulated in individuals with *C. trachomatis* infection and *SOCS3* and *IL10* were upregulated in individuals with TS and TP, indicative of inflammatory regulation. Upregulation of *IL10* expression has previously been reported in individuals with active trachoma and a genetic polymorphism in *IL10* was associated with trachomatous scarring (Mozzato-Chamay et al., 2000; Burton et al., 2004; Natividad et al., 2005; Faal et al., 2006; Skwor et al., 2008). However, the role of Tregs and IL-10 in trachoma remains unclear; dampening inflammation might result in less tissue damage, but conversely it might also prolong survival of *C. trachomatis* (Zhang et al., 2009).

*MMP9, MMP12, TGF*β*1*, and *PDGF* were significantly upregulated in individuals with *C. trachomatis* infection. *MMP7, MMP9*, and *MMP12* were upregulated in individuals with TP, *MMP9 was* upregulated in TF and *FGF2* and *PDGF* were upregulated in individuals with TS. *MMP7* was downregulated in infected individuals. *MMP7* has consistently been found to be upregulated in scarring trachoma and trichiasis alongside *MMP9* and *MMP12* but there is less evidence for a role of *MMP7* in active trachoma (Holland et al., 2010; Burton et al., 2011b, 2015; Hu et al., 2012). Degradation of the extracellular matrix and basement membrane by MMP9 and MMP12, in part derived from macrophage and neutrophil degranulation (Shapiro et al., 1993; Kjeldsen et al., 1994), facilitates connective tissue remodeling and leukocyte migration into the epithelium (Ozdemir et al., 1999; Zeng et al., 1999; Misko et al., 2002). Whilst MMPs are an essential part of the wound healing process (Nwomeh et al., 1998; Wolf et al., 2017), MMP9 overexpression has been linked to aggressive contractile scarring in proliferative vitroretinopathy and failure of rat corneal cells to re-epithelialize following corneal injury (Fini et al., 1996; Kon et al., 1998).

*SPARCL1* was down-regulated in individuals with *C. trachomatis* infection, TF and TP, consistent with previous studies (Hu et al., 2012; Burton et al., 2015). *SPARCL1* codes for a secreted glycoprotein which regulates extracellular matrix (ECM) and cell-matrix adhesion (Girard and Springer, 1996) SPARCL1 inhibits cell proliferation and migration and reduced *SPARCL1* expression has been linked to cancer metastases (Nelson et al., 1998; Sullivan and Sage, 2004). In a murine corneal injury model, knock-out of SPARCL1 led to excessive accumulation of collagen type IV from myofibroblasts and irregular fibrotic ECM formation (Chaurasia et al., 2013). Addition of exogenous SPARCL1 rescued the wild type phenotype with a cessation of collagen IV production, replacement with collagen I from keratinocytes and restoration of normal ECM architecture. Consistent with this, immunohistochemistry and histology studies using tissue from individuals with scarring trachoma have demonstrated a reduction in collagens I and III, an increase in collagens IV and V and progressive disorganization of collagen fibers in scarring trachoma relative to healthy conjunctival tissue (Abu El-Asrar et al., 1998; Hu et al., 2013b; Derrick et al., 2016b). Genes that were most strongly associated with active trachoma in the absence of *C. trachomatis* infection were enriched for pathways of leukocyte transendothelial migration, TNF receptors, MMP activation, collagen formation and assembly (Table 6). Active trachoma signs often persist after the clearance of *C. trachomatis* infection (Grassly et al., 2008; Burton et al., 2011a,c; Lee et al., 2014) and inflammation is linked to progressive scarring (Ramadhani et al., 2016a). We found that TP but not infection was associated with TS in the multivariable model (Table 3). These results suggest that the inflammatory phenotype following the clearance of *C. trachomatis* infection is characterized by collagen formation and assembly, MMP-mediated matrix remodeling and leukocyte influx, and that these pathways might be causative factors in the scarring process.

We found significantly reduced expression of *MUC1, MUC4, MUC5AC*, and *MUC7* in individuals with *C. trachomatis* infection, and *MUC5AC* expression was reduced in those with TP. MUC1 and MUC4 are primarily expressed by apical cells of the stratified epithelia, MUC7 by lacrimal gland epithelia and MUC5AC by goblet cells (Gipson and Argueso, 2003). *MUC7* expression was also significantly reduced in females relative to males. *MUC5AC* and *MUC7* down-regulation has previously been reported in individuals with trachomatous trichiasis, however expression of *MUC1* and *MUC4* was increased in this later stage of disease (Burton et al., 2011b). IL-22 stimulates the upregulation of MUC4 and MUC1, therefore, one might expect a corresponding increase in mucin expression in infected individuals (Turner et al., 2013). This inconsistency and the broad reduction in mucin expression during infection may reflect a loss of epithelial homeostasis and a potential reduction in barrier function. This in turn could result in increased access of bacteria to the epithelial cell surface, promoting inflammation. In patients with ulcerative colitis, reduction of goblet cells, mucin production and microbial diversity were identified as causative factors in disease etiology (Alipour et al., 2016). Microbial dysbiosis can itself be caused by inflammation, creating a positive feedback loop (Winter et al., 2013). Decreased diversity has previously been found in the conjunctival microbiome of individuals with scarring trachoma relative to healthy controls (Zhou et al., 2014). Furthermore, penetration of the mucin barrier and contact between epithelial cells and bacteria was demonstrated in ulcerative colitis patients with acute inflammation, whereas the mucin barrier of healthy controls was impenetrable (Johansson et al., 2014). It is tempting to speculate that reduced barrier function resulting from inflammation and loss of mucins causes microbial dysbiosis and contact between bacteria and epithelial cells, leading to persistent conjunctival inflammation, which in turn contributes to trachomatous scarring.

Consistent with our previous studies in various clinical stages of trachoma (Burton et al., 2011b,c, 2012, 2015; Hu et al., 2012), the antimicrobial peptide S100A7 was upregulated in individuals with TP and was marginally associated with *C. trachomatis* infection. S100A7 is secreted by epithelial cells in response to microbial challenge and expression can also be stimulated by IL-22 (Wolk et al., 2006). It is chemotactic for neutrophils and interferes with pathogen membrane permeability and enzyme function (Ganz, 2003; Guilhelmelli et al., 2013). The upregulation of S100A7 in various clinical stages of trachoma could support the loss of epithelial barrier function hypothesis described above and could contribute to chronic inflammation.

There was differential expression of several cell cycle genes (*CD53, CDK13, CDC25C, PHYH, TYMS, TTK, TP53*) in individuals with *C. trachomatis* infection and/or TP (Table 5). These expression changes could reflect regulation of the cell cycle in response to infection or inflammation, such as the proliferation or contraction of infiltrating inflammatory cells and cells effecting the wound healing response. Studies have reported that progression of the cell cycle is slow in cells infected with *C. trachomatis* (Kun et al., 2013; Elwell et al., 2016) and *C. trachomatis* is known to interfere with host cell apoptosis as a survival mechanism (Fischer et al., 2004; Ying et al., 2007; Siegl et al., 2014). Cluster 1 was enriched for genes in apoptosis pathways, however the combined expression of this cluster was not associated with clinical phenotypes or infection.

## Conclusions

**Figure 3:**
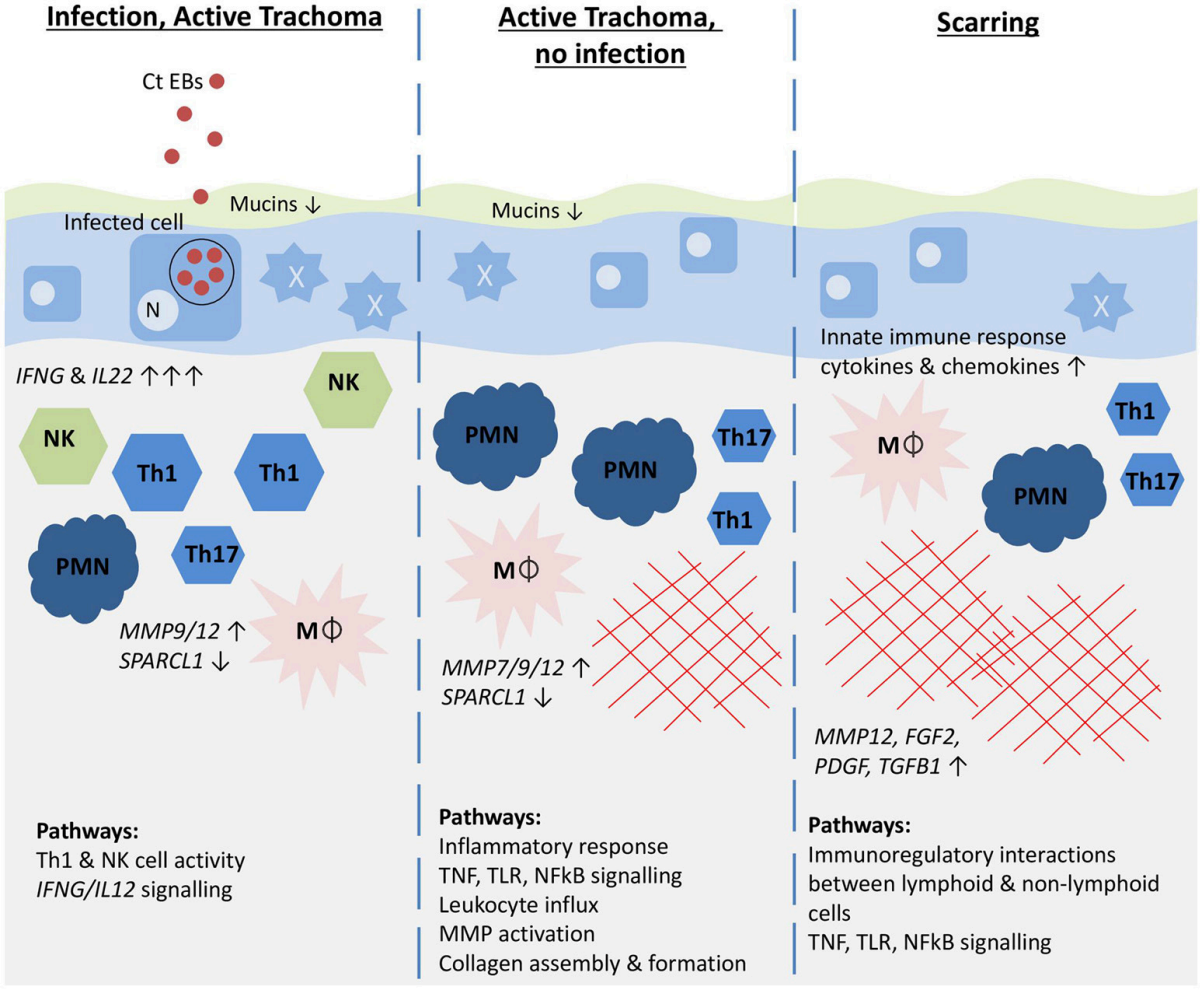
Graphical summary illustrating the host immune pathways hypothesized to be associated with different trachoma phenotypes. Genes most strongly associated with each phenotype are shown in italics with the direction and strength of expression illustrated by arrows. Pathways that were most enriched for each phenotype are shown. Ct EBs, *C. trachomatis* elementary bodies; N, epithelial cell nucleus; NK, Natural killer cells; Th1, Th1 T cells; Th17, Th17 T cells; PMN, neutrophil; Mϕ, macrophage; red cross-hatching, fibrosis. The mucus layer is shown in green, the epithelial cell layer in blue, and the stroma in beige.

Our results, illustrated in a graphical summary (Figure 3), suggest that *IFNG* and *IL22* were acutely upregulated in response to *C. trachomatis* infection and expression appeared to be reduced following the clearance of infection, despite the persistence of inflammation. This might suggest that the sources of these cytokines are important in the clearance of infection and that the inflammatory phenotype differs once infection has been cleared, shifting away from a Th1/NK cell dominated response. The upregulation of Th17 cell-associated cytokines in infection and TP phenotypes implies the involvement of Th17 cells, although it remains unclear whether they contribute more to protection or pathology. Epithelial cells appear to be highly active, with possible loss of epithelial homeostasis and barrier function resulting in loss of mucins and a pro-inflammatory anti-microbial phenotype, perhaps exacerbating inflammation. The upregulation of growth factors and MMPs, derived in part from infiltrating macrophages and neutrophils, and the down-regulation of SPARCL1 are likely to contribute to matrix remodeling, collagen deposition and conjunctival scarring in the post-infection inflammatory phase. Females were more susceptible to TP and TS and although this was not associated with a significant difference in infection prevalence, there was some evidence of increased *IFNG* related pathway activity in females. A caveat of this study is that gene expression may not correlate directly to protein expression and this will require further work. Despite this, we anticipate that longitudinal investigation of these transcriptional signatures and relation to infection and ongoing clinical signs will enhance our understanding of their relative contributions to protection and pathology.

## Author contributions

Substantial contributions to the conception: MB, MH, DCM, and RB. Design of the work: MB, AR, MH, PM, TM and CR. Analysis: AR, MB, DM, MH, TD, and DJ. Interpretation of data for the work: AR, MB, MH, and TD. Drafting the work: AR and MB. Revising it critically for important intellectual content: AR, MB, MH, TD, DCM, RB, DM, CR, PM, TM, and DJ. Final approval of the version to be published: AR, MB, MH, TD, DCM, RB, DM, CR, PM, TM, and DJ. Agreement to be accountable for all aspects of the work in ensuring that questions related to the accuracy or integrity of any part of the work are appropriately investigated and resolved: AR, MB, MH, TD, DCM, RB, DM, CR, PM, TM, and DJ.

### Conflict of interest statement

The authors declare that the research was conducted in the absence of any commercial or financial relationships that could be construed as a potential conflict of interest.
