# Supplementary material for: Immunofibrogenic Gene Expression Patterns in Tanzanian Children with Ocular Chlamydia trachomatis Infection, Active Trachoma and Scarring: Baseline Results of a 4-Year Longitudinal Study
Source: Front Cell Infect Microbiol. 2017 Sep 15;7:406. doi: 10.3389/fcimb.2017.00406 (PMC5605569; doi:10.3389/fcimb.2017.00406)
Supplement: Supplementary file 1 [file Table1.DOCX]

**Supplementary Table 1: Comparison of conjunctival gene expression in the presence of *C. trachomatis* plasmid, follicular inflammation (TF), papillary inflammation (TP) and scarring trachoma, adjusted for age and sex. N – number of individuals with detectable expressions. FC – fold change. P-value. Using the Benjamini and Hochberg approach only tests with a p-value below 0.0350 have a False Discovery Rate of <5%.**

|  |  | **Infected vs not** | | **TF vs no TF** | | **TP vs no TP** | | **TS vs no TS** | |
| --- | --- | --- | --- | --- | --- | --- | --- | --- | --- |
| **Target** | **N** | **FC** | **p-value** | **FC** | **p-value** | **FC** | **p-value** | **FC** | **p-value** |
| **Antimicrobial Peptides** | | | | | | | | | |
| Defensin, beta 4B,defensin, beta 4A (*DEFB4A*) | 494 | **1.57** | **0.0044** | **1.31** | **0.0330** | **1.96** | **4.70E-08** | **1.33** | **0.0258** |
| Psoriasin-1 (*S100A7*) | 492 | **3.43** | **5.81E-07** | **3.24** | **1.58E-09** | **4.30** | **3.88E-14** | **1.91** | **0.0012** |
| **Cell Cycle** | | | | | | | | | |
| Catenin (cadherin-associated protein), delta 2 (*CTNND2*) | 327 | 0.75 | 0.1967 | 0.89 | 0.5377 | 0.79 | 0.2123 | 0.95 | 0.7887 |
| CD53 molecule (*CD53*) | 494 | **1.24** | **0.0003** | **1.15** | **0.0022** | **1.24** | **2.78E-06** | **1.14** | **0.0049** |
| Cyclin-dependent kinase 13 (*CDK13*) | 494 | **0.87** | **0.0029** | **0.91** | **0.0080** | **0.86** | **4.29E-05** | 0.97 | 0.4573 |
| M-phase inducer phosphatase 3 (*CDC25C*) | 485 | **1.53** | **1.88E-07** | **1.36** | **2.03E-06** | **1.29** | **7.11E-05** | 1.13 | 0.0548 |
| Phytanoyl-coa 2-hydroxylase (*PHYH*) | 494 | **0.80** | **7.36E-07** | **0.85** | **6.84E-06** | **0.87** | **0.0001** | 0.96 | 0.2410 |
| Sun Domain Family, Member 6 (*NSUN6*) | 494 | 0.98 | 0.4884 | **0.94** | **0.0235** | 0.96 | 0.1036 | 1.02 | 0.5273 |
| Thymidylate synthetase (*TYMS*) | 494 | **1.82** | **8.78E-25** | **1.41** | **2.79E-13** | **1.41** | **5.38E-13** | **1.13** | **0.0144** |
| TTK protein kinase (*TTK*) | 493 | **1.51** | **6.25E-10** | **1.37** | **1.50E-09** | **1.34** | **3.26E-08** | **1.21** | **0.0005** |
| Tumor protein p53 (*TP53*) | 494 | 0.95 | 0.2391 | **0.93** | **0.0281** | **0.87** | **9.41E-06** | 0.99 | 0.7425 |
| **Cytokines/Chemokines** | | | | | | | | | |
| Chemokine ligand 2 (*CCL2*) | 490 | **4.29** | **3.77E-21** | **2.00** | **4.32E-08** | **2.39** | **3.53E-12** | **1.83** | **2.73E-06** |
| Chemokine ligand 5 (*CXCL5*) | 493 | 1.22 | 0.1911 | **1.63** | **6.09E-05** | **2.08** | **1.55E-09** | **1.88** | **2.64E-07** |
| Chemokine ligand 13 (*CXCL13*) | 491 | **3.48** | **2.10E-13** | **2.76** | **5.04E-14** | **3.11** | **2.55E-17** | **1.62** | **0.0006** |
| Chemokine ligand 18 (*CCL18*) | 488 | **3.16** | **9.96E-10** | **2.33** | **1.82E-08** | **3.59** | **3.75E-18** | 1.37 | 0.0411 |
| Chemokine ligand 20 (*CCL20*) | 493 | **1.59** | **1.86E-05** | **1.68** | **1.24E-09** | **1.46** | **8.91E-06** | **1.49** | **4.27E-06** |
| Chemokine receptor 6 (*CCR6*) | 493 | 1.29 | 0.0493 | **1.45** | **0.0003** | 1.12 | 0.2586 | 1.17 | 0.1329 |
| Colony stimulating factor 2 (*CSF2*) | 489 | **2.79** | **1.15E-22** | **1.68** | **1.93E-09** | **1.53** | **1.10E-06** | **1.32** | **0.0015** |
| Colony stimulating factor 3 (*CSF3*) | 493 | 1.05 | 0.7614 | 1.22 | 0.0805 | **1.47** | **0.0009** | **1.62** | **3.30E-05** |
| Forkhead box P3 (*FOXP3*) | 491 | **1.25** | **0.0004** | 1.08 | 0.1071 | 1.07 | 0.1985 | 0.95 | 0.2866 |
| Indoleamine 2,3-dioxygenase 1 (*IDO1*) | 494 | **2.18** | **2.74E-12** | **1.69** | **3.79E-09** | **1.73** | **7.00E-10** | **1.47** | **1.99E-05** |
| Interferon gamma(*IFNγ*) | 491 | **8.81** | **2.54E-57** | **2.74** | **1.64E-17** | **2.28** | **6.80E-12** | **1.56** | **0.0003** |
| Interleukin 1 beta(*IL1β*) | 493 | **2.05** | **1.11E-08** | **1.64** | **6.93E-07** | **2.07** | **1.42E-13** | **1.65** | **7.84E-07** |
| Interleukin 6 (*IL6*) | 493 | **2.00** | **2.11E-07** | **1.35** | **0.0056** | **1.51** | **0.0001** | **1.43** | **0.0010** |
| Interleukin 8 (*IL8*) | 493 | **1.49** | **1.92E-05** | **1.23** | **0.0054** | **1.51** | **2.73E-08** | **1.38** | **2.27E-05** |
| Interleukin 10 (*IL10*) | 492 | **2.33** | **2.73E-16** | **1.78** | **2.63E-12** | **1.97** | **1.40E-16** | **1.50** | **1.91E-06** |
| Interleukin 12 beta (*IL12β*) | 480 | **3.99** | **2.91E-30** | **1.92** | **1.38E-10** | **1.84** | **2.20E-09** | **1.35** | **0.0045** |
| Interleukin 13 (*IL13*) | 214 | 1.38 | 0.1496 | 1.37 | 0.1247 | 1.02 | 0.9098 | 0.83 | 0.3233 |
| Interleukin 17A (*IL17A*) | 492 | **3.23** | **4.00E-18** | **2.11** | **6.23E-12** | **2.37** | **1.15E-15** | **1.57** | **5.34E-05** |
| Interleukin 19 (*IL19*) | 493 | **3.21** | **1.35E-13** | **2.20** | **3.83E-10** | **3.26** | **5.97E-22** | **1.63** | **0.0001** |
| Interleukin 21 (*IL21*) | 480 | **4.89** | **2.08E-24** | **3.09** | **2.34E-19** | **2.70** | **3.82E-15** | **1.87** | **1.99E-06** |
| Interleukin 22 (*IL22*) | 427 | **8.01** | **4.91E-33** | **2.66** | **1.26E-10** | **2.50** | **1.81E-09** | **1.81** | **0.0002** |
| Interleukin 23A (*IL23A*) | 492 | **2.38** | **3.16E-17** | **1.68** | **4.06E-10** | **2.04** | **1.15E-18** | **1.36** | **0.0002** |
| Interleukin 33 (*IL33*) | 494 | **1.26** | **0.0121** | 1.04 | 0.6076 | 1.02 | 0.7717 | **1.28** | **0.0007** |
| Prostaglandin-endoperoxide synthase 2 (*PTGS2*) | 494 | **1.37** | **0.0054** | **1.24** | **0.0175** | **1.52** | **3.22E-06** | **1.39** | **0.0003** |
| Tumor necrosis factor (*TNF*) | 492 | **1.53** | **0.0002** | **1.49** | **1.02E-05** | **1.25** | **0.0139** | **1.26** | **0.0123** |
| **EMT Markers** | | | | | | | | | |
| Alpha smooth muscle actin  (*ACTA2*) | 492 | **1.28** | **0.0002** | 1.10 | 0.0729 | 0.97 | 0.5791 | **1.14** | **0.0118** |
| Epithelial cadherin (*CDH1*) | 493 | **0.73** | **4.34E-08** | **0.84** | **6.89E-05** | **0.84** | **0.0002** | 0.98 | 0.7421 |
| Cadherin 1, type 1, E-cadherin (epithelial) (CDH1) | 494 | **0.69** | **2.16E-09** | **0.82** | **9.65E-05** | **0.83** | **0.0002** | 0.97 | 0.5543 |
| Neuronal cadherin (*CDH2*) | 482 | 0.98 | 0.8961 | 0.86 | 0.1328 | **0.76** | **0.0049** | 1.09 | 0.4063 |
| S100 calcium binding protein A4 (*S100A4*) | 493 | **0.39** | **1.39E-32** | **0.58** | **8.58E-17** | **0.56** | **1.66E-19** | 0.93 | 0.2657 |
| Vimentin (*VIM*) | 494 | **1.57** | **3.65E-20** | **1.20** | **2.99E-06** | **1.19** | **1.55E-05** | **1.11** | **0.0073** |
| **Matrix Modifiers** | | | | | | | | | |
| Connective tissue growth factor (*CTGF-1*) | 493 | 1.08 | 0.4138 | 0.91 | 0.2104 | **0.83** | **0.0117** | 0.94 | 0.3853 |
| Connective tissue growth factor (*CTGF-2*) | 494 | 1.04 | 0.6659 | **0.84** | **0.0218** | **0.81** | **0.0052** | 0.87 | 0.0603 |
| Fibroblast growth factor 2 (basic) (*FGF2*) | 399 | **1.54** | **0.0021** | 1.12 | 0.3424 | 1.25 | 0.0569 | **1.51** | **0.0005** |
| Matrix metallopeptidase 7 (*MMP7*) | 493 | **0.42** | **1.38E-13** | 0.84 | 0.0674 | 1.05 | 0.6140 | 0.99 | 0.9536 |
| Matrix metallopeptidase 9 (*MMP9*) | 493 | **2.51** | **8.58E-15** | **2.11** | **1.91E-15** | **2.11** | **2.14E-15** | **1.51** | **2.08E-05** |
| Matrix metallopeptidase 12 (*MMP12*) | 493 | **2.85** | **3.14E-16** | **2.17** | **3.60E-14** | **2.23** | **3.53E-15** | **1.62** | **4.33E-06** |
| Platelet-derived growth factor beta polypeptide (*PDGFβ*) | 492 | **1.53** | **1.88E-11** | **1.22** | **9.88E-05** | **1.25** | **1.59E-05** | **1.24** | **3.14E-05** |
| SPARC-like 1 (hevin) (*SPARCL1*) | 484 | **0.22** | **1.74E-10** | **0.23** | **1.04E-14** | **0.21** | **1.54E-16** | **0.52** | **0.0009** |
| Transforming growth factor, beta 1 (*TGFβ1*) | 493 | **1.25** | **3.23E-06** | **1.10** | **0.0156** | 1.08 | 0.0572 | **1.11** | **0.0077** |
| Transforming growth factor, beta 2 (*TGFβ2*) | 492 | 0.89 | 0.2044 | 0.93 | 0.3235 | 0.93 | 0.3581 | 1.05 | 0.5253 |
| **Microbiota Response** | | | | | | | | | |
| Arachidonate 5-lipoxygenase (*ALOX5*) | 494 | **0.62** | **1.44E-20** | **0.77** | **1.62E-10** | **0.81** | **1.06E-06** | 0.96 | 0.3789 |
| B-cell CLL/lymphoma 2 (*BCL2*) | 494 | **1.11** | **0.0217** | 1.02 | 0.6071 | 0.99 | 0.7735 | **1.09** | **0.0241** |
| CD40 molecule, TNF receptor superfamily member 5 (*CD40*) | 494 | **1.24** | **0.0004** | 1.05 | 0.2999 | 1.05 | 0.3513 | 1.08 | 0.1334 |
| Dual oxidase 2 (*DUOX2*) | 494 | **1.32** | **0.0022** | **1.19** | **0.0180** | **1.40** | **2.70E-06** | **1.19** | **0.0202** |
| Tumor necrosis factor receptor superfamily, member 1A (*TNFRSF1A*) | 494 | **0.76** | **0.0046** | **0.82** | **0.0105** | 0.88 | 0.0995 | 1.01 | 0.8651 |
| Tumor necrosis factor receptor superfamily, member 1B (*TNFRSF1B*) | 494 | 1.05 | 0.5043 | 1.01 | 0.8897 | **1.14** | **0.0190** | **1.13** | **0.0337** |
| V-rel avian reticuloendotheliosis viral oncogene homolog (*REL*) | 494 | 1.05 | 0.2782 | 1.02 | 0.5318 | 1.08 | 0.0413 | 1.07 | 0.0657 |
| **Mucin** | | | | | | | | | |
| Mucin 1, cell surface associated (*MUC1*) | 494 | **0.80** | **0.0001** | **0.89** | **0.0110** | 0.95 | 0.2538 | 1.00 | 0.9778 |
| Mucin 4, cell surface associated (*MUC4*) | 494 | **0.73** | **1.17E-05** | 0.91 | 0.1154 | 1.02 | 0.7338 | 1.04 | 0.4983 |
| Mucin 5AC, oligomeric mucus/gel-forming (*MUC5AC*) | 494 | **0.41** | **2.69E-10** | **0.50** | **6.86E-10** | **0.48** | **2.05E-11** | 0.96 | 0.7406 |
| Mucin 7, secreted (*MUC7*) | 494 | **0.33** | **1.76E-11** | **0.52** | **6.24E-07** | **0.49** | **8.02E-08** | **0.74** | **0.0248** |
| **NK Cell Markers** | | | | | | | | | |
| *CD247* molecule (*CD247*) | 493 | **1.93** | **6.19E-22** | **1.37** | **1.65E-08** | **1.28** | **1.14E-05** | 1.11 | 0.0776 |
| Natural cytotoxicity triggering receptor 1 (*NCR1*) | 492 | **2.50** | **1.13E-30** | **1.50** | **1.27E-09** | **1.38** | **1.14E-06** | **1.28** | **0.0003** |
| Neural cell adhesion molecule 1 (*NCAM1*) | 493 | **1.50** | **2.08E-06** | 0.90 | 0.1171 | **0.83** | **0.0080** | 1.10 | 0.1742 |
| **Pattern Recognition Receptors** | | | | | | | | | |
| Nucleotide-binding oligomerization domain containing 2 (*NOD2*) | 491 | 1.13 | 0.1847 | 1.04 | 0.6135 | **1.19** | **0.0174** | 1.17 | 0.0377 |
| Toll-like receptor 2 (*TLR2*) | 493 | 0.89 | 0.1577 | 1.01 | 0.8602 | 1.03 | 0.6513 | **1.24** | **0.0018** |
| Toll-like receptor 4 (*TLR4*) | 493 | 1.13 | 0.1092 | **1.15** | **0.0218** | **1.26** | **0.0002** | **1.26** | **0.0002** |
| **Regulators/Signalling Pathways** | | | | | | | | | |
| CD274 molecule (*CD274*) | 493 | **2.99** | **5.54E-29** | **1.73** | **1.58E-11** | **1.92** | **3.44E-16** | **1.43** | **1.41E-05** |
| Chromodomain helicase DNA binding protein 8 (*CHD8*) | 494 | 0.97 | 0.4299 | 0.95 | 0.0481 | 0.98 | 0.4673 | 1.02 | 0.4319 |
| COMM domain containing 6 (*COMMD6*) | 493 | 0.87 | 0.1506 | 0.93 | 0.3440 | 0.92 | 0.2886 | 0.97 | 0.6802 |
| Hematopoietically expressed homeobox (*HHEX*) | 493 | **1.26** | **0.0004** | **1.35** | **1.19E-08** | **1.15** | **0.0083** | **1.14** | **0.0177** |
| IKAROS family zinc finger 1 (Ikaros) (*IKZF1*) | 494 | **1.45** | **2.24E-13** | **1.24** | **2.04E-07** | **1.16** | **0.0002** | **1.10** | **0.0232** |
| Marginal zone B and B1 cell-specific protein (*MZB1*) | 493 | **2.44** | **1.08E-12** | **1.65** | **5.78E-07** | **1.64** | **1.03E-06** | 1.15 | 0.1880 |
| Myeloid differentiation primary response 88 (*MYD88*) | 494 | 1.00 | 0.8889 | 0.97 | 0.2443 | 1.02 | 0.3767 | **1.07** | **0.0156** |
| Nuclear factor of kappa light polypeptide gene enhancer in B-cells 1 (*NFKB1*) | 494 | **1.13** | **0.0005** | 1.03 | 0.3739 | **1.07** | **0.0168** | **1.06** | **0.0341** |
| Phosphatidylinositol-3,4,5-trisphosphate-dependent Rac exchange factor 2 (*PREX2v1*) | 462 | **1.28** | **0.0335** | 1.02 | 0.7928 | 0.89 | 0.2381 | 0.90 | 0.2483 |
| Phosphatidylinositol-3,4,5-trisphosphate-dependent Rac exchange factor 2 (*PREX2v2*) | 445 | 1.09 | 0.4465 | 1.10 | 0.3299 | 1.09 | 0.3703 | 0.88 | 0.2025 |
| Ras homolog family member H (*RHOH*) | 494 | **1.63** | **4.48E-14** | **1.38** | **4.78E-10** | **1.26** | **8.00E-06** | **1.13** | **0.0271** |
| SAM domain, SH3 domain and nuclear localization signals 1 (*SAMSN1*) | 494 | **1.57** | **2.47E-10** | **1.35** | **8.85E-08** | **1.48** | **2.48E-12** | **1.17** | **0.0067** |
| Serglycin (*SRGN*) | 494 | 1.05 | 0.5256 | 1.07 | 0.2821 | **1.24** | **0.0006** | **1.17** | **0.0122** |
| Serpin peptidase inhibitor B3 (*SERPINB3*) | 493 | **3.43** | **2.87E-16** | **1.83** | **7.66E-07** | **2.45** | **8.52E-14** | **1.72** | **1.21E-05** |
| Serpin peptidase inhibitor clade B member 4, (*SERPINB4*) | 406 | **5.93** | **1.95E-11** | **2.32** | **0.0002** | **3.80** | **8.81E-10** | **1.80** | **0.0089** |
| Signal transducer and activator of transcription 1 (*STAT1*) | 493 | **2.13** | **1.90E-31** | **1.38** | **2.63E-09** | **1.40** | **5.78E-10** | **1.25** | **6.72E-05** |
| Signal transducer and activator of transcription 3 (*STAT3*) | 493 | 1.02 | 0.6831 | 1.00 | 0.9047 | 1.06 | 0.1165 | **1.14** | **0.0003** |
| Signal transducer and activator of transcription 4 (*STAT4*) | 493 | **1.88** | **2.22E-22** | **1.25** | **3.32E-05** | **1.21** | **0.0005** | **1.12** | **0.0312** |
| Suppressor of cytokine signalling 1 (*SOCS1*) | 493 | **2.34** | **4.52E-27** | **1.56** | **6.81E-12** | **1.48** | **1.55E-09** | **1.26** | **0.0006** |
| Suppressor of cytokine signalling 3 (*SOCS3*) | 493 | **1.53** | **3.68E-05** | **1.44** | **6.37E-06** | **1.71** | **2.48E-11** | **1.53** | **2.02E-07** |
| T-box 21 (*TBX21*) | 493 | **2.42** | **7.94E-36** | **1.57** | **2.30E-14** | **1.40** | **1.79E-08** | **1.22** | **0.0011** |
| Ubiquitin specific peptidase 6 (Tre-2 oncogene) (*USP6*) | 182 | 0.72 | 0.2233 | **0.61** | **0.0298** | 0.66 | 0.0766 | 0.66 | 0.0716 |
