# Supplementary material for: Immunofibrogenic Gene Expression Patterns in Tanzanian Children with Ocular Chlamydia trachomatis Infection, Active Trachoma and Scarring: Baseline Results of a 4-Year Longitudinal Study
Source: Front Cell Infect Microbiol. 2017 Sep 15;7:406. doi: 10.3389/fcimb.2017.00406 (PMC5605569; doi:10.3389/fcimb.2017.00406)
Supplement: Supplementary file 2 [file Table2.DOCX]

**Supplementary Table 2a. Network co-expression analysis of the filtered dataset using Miru and Markov clustering revealed five clusters of co-expressed genes**.

| **Cluster 1** | | **Cluster 2** | | **Cluster 3** | | **Cluster 4** | | **Cluster 5** | |
| --- | --- | --- | --- | --- | --- | --- | --- | --- | --- |
| **Genes** | **Connections** | **Genes** | **Connections** | **Genes** | Connections | **Genes** | **Connections** | **Genes** | **Connections** |
| MYD88 | 13 | IKZF1 | 11 | STAT1 | 6 | SRGN | 6 | IL10 | 3 |
| REL | 13 | VIM | 11 | CD247 | 5 | TLR4 | 4 | IL17A | 3 |
| NFKB1 | 12 | CD53 | 10 | STAT4 | 5 | TNFRSF1B | 4 | IL21 | 3 |
| CHD8 | 11 | SAMSN1 | 10 | IFNG | 2 | IL1B | 3 | CXCL13 | 1 |
| BCL2 | 10 | RHOH | 6 | NCR1 | 2 | PTGS2 | 3 | MMP12 | 1 |
| TP53 | 9 | TBX21 | 6 | PDGFB | 1 | SOCS3 | 3 |  |  |
| CDK13 | 8 | TYMS | 6 |  |  |  |  |  |  |
| NSUN6 | 8 | CD274 | 4 |  |  |  |  |  |  |
| PHYH | 6 | HHEX | 3 |  |  |  |  |  |  |
| CD40 | 5 | TTK | 1 |  |  |  |  |  |  |
| ALOX5 | 1 |  |  |  |  |  |  |  |  |

**Supplementary Table 2b. Pathway enrichment analysis of genes in each cluster against background, using ConsensusPathDB.** The first six most enriched pathways (P < 0.05) are shown.

| **pathway name** | **set size^a^** | **candidates^b^** | **p-value** | **q-value** | **pathway source** |
| --- | --- | --- | --- | --- | --- |
|  |  |  |  |  |  |
| **Cluster 1** |  |  |  |  |  |
| Neurotrophin signaling pathway | [121(3)](http://cpdb.molgen.mpg.de/CPDB/showSetDetails?sp=p&st=0) | [3 (100.0%)](http://cpdb.molgen.mpg.de/CPDB/showSetDetails?sp=p&st=0) | 0.00217 | 0.0286 | KEGG |
| Apoptosis Modulation and Signaling | [92(6)](http://cpdb.molgen.mpg.de/CPDB/showSetDetails?sp=p&st=1) | [4 (66.7%)](http://cpdb.molgen.mpg.de/CPDB/showSetDetails?sp=p&st=1) | 0.00296 | 0.0286 | Wikipathways |
| DNA Damage Response (only ATM dependent) | [110(4)](http://cpdb.molgen.mpg.de/CPDB/showSetDetails?sp=p&st=5) | [3 (75.0%)](http://cpdb.molgen.mpg.de/CPDB/showSetDetails?sp=p&st=5) | 0.00798 | 0.0286 | Wikipathways |
| IL1 | [56(4)](http://cpdb.molgen.mpg.de/CPDB/showSetDetails?sp=p&st=6) | [3 (75.0%)](http://cpdb.molgen.mpg.de/CPDB/showSetDetails?sp=p&st=6) | 0.00798 | 0.0286 | NetPath |
| Downstream signaling events of B Cell Receptor (BCR) | 147(4) | 3 (75.0%) | 0.00798 | 0.0286 | Reactome |
| Signaling by the B Cell Receptor (BCR) | 242(4) | 3 (75.0%) | 0.00798 | 0.0286 | Reactome |
| Viral carcinogenesis | [203(4)](http://cpdb.molgen.mpg.de/CPDB/showSetDetails?sp=p&st=7) | [3 (75.0%)](http://cpdb.molgen.mpg.de/CPDB/showSetDetails?sp=p&st=7) | 0.00798 | 0.0286 | KEGG |
| Prostate cancer | [89(4)](http://cpdb.molgen.mpg.de/CPDB/showSetDetails?sp=p&st=4) | [3 (75.0%)](http://cpdb.molgen.mpg.de/CPDB/showSetDetails?sp=p&st=4) | 0.00798 | 0.0286 | KEGG |
|  |  |  |  |  |  |
| **Cluster 2** |  |  |  |  |  |
| Retinoblastoma (RB) in Cancer | [89(3)](http://cpdb.molgen.mpg.de/CPDB/showSetDetails?sp=p&st=0) | [2 (66.7%)](http://cpdb.molgen.mpg.de/CPDB/showSetDetails?sp=p&st=0) | 0.0418 | 0.155 | Wikipathways |
|  |  |  |  |  |  |
| **Cluster 3** |  |  |  |  |  |
| Th1 and Th2 cell differentiation | [92(7)](http://cpdb.molgen.mpg.de/CPDB/showSetDetails?sp=p&st=0) | [4 (57.1%)](http://cpdb.molgen.mpg.de/CPDB/showSetDetails?sp=p&st=0) | 0.000344 | 0.0235 | KEGG |
| SHP2 signaling | [59(4)](http://cpdb.molgen.mpg.de/CPDB/showSetDetails?sp=p&st=1) | [3 (75.0%)](http://cpdb.molgen.mpg.de/CPDB/showSetDetails?sp=p&st=1) | 0.00102 | 0.0235 | PID |
| Downstream signaling in naïve CD8+ T cells | [68(4)](http://cpdb.molgen.mpg.de/CPDB/showSetDetails?sp=p&st=2) | [3 (75.0%)](http://cpdb.molgen.mpg.de/CPDB/showSetDetails?sp=p&st=2) | 0.00102 | 0.0235 | PID |
| IL12-mediated signaling events | [64(10)](http://cpdb.molgen.mpg.de/CPDB/showSetDetails?sp=p&st=3) | [4 (40.0%)](http://cpdb.molgen.mpg.de/CPDB/showSetDetails?sp=p&st=3) | 0.00193 | 0.0333 | PID |
| Natural killer cell mediated cytotoxicity | [135(5)](http://cpdb.molgen.mpg.de/CPDB/showSetDetails?sp=p&st=4) | [3 (60.0%)](http://cpdb.molgen.mpg.de/CPDB/showSetDetails?sp=p&st=4) | 0.00247 | 0.0341 | KEGG |
| IL12 signaling mediated by STAT4 | [32(6)](http://cpdb.molgen.mpg.de/CPDB/showSetDetails?sp=p&st=5) | [3 (50.0%)](http://cpdb.molgen.mpg.de/CPDB/showSetDetails?sp=p&st=5) | 0.00479 | 0.0431 | PID |
| IFN gamma signaling pathway | [6(2)](http://cpdb.molgen.mpg.de/CPDB/showSetDetails?sp=p&st=6) | [2 (100.0%)](http://cpdb.molgen.mpg.de/CPDB/showSetDetails?sp=p&st=6) | 0.005 | 0.0431 | BioCarta |
| IFN gamma signaling | [6(2)](http://cpdb.molgen.mpg.de/CPDB/showSetDetails?sp=p&st=7) | [2 (100.0%)](http://cpdb.molgen.mpg.de/CPDB/showSetDetails?sp=p&st=7) | 0.005 | 0.0431 | INOH |
|  |  |  |  |  |  |
| **Cluster 4** |  |  |  |  |  |
| TNF signaling pathway | [110(14)](http://cpdb.molgen.mpg.de/CPDB/showSetDetails?sp=p&st=0) | [4 (28.6%)](http://cpdb.molgen.mpg.de/CPDB/showSetDetails?sp=p&st=0) | 0.00837 | 0.191 | KEGG |
| Nanomaterial induced inflammasome activation | [3(3)](http://cpdb.molgen.mpg.de/CPDB/showSetDetails?sp=p&st=1) | [2 (66.7%)](http://cpdb.molgen.mpg.de/CPDB/showSetDetails?sp=p&st=1) | 0.0145 | 0.191 | Wikipathways |
| TLR ECSIT MEKK1 JNK | [27(3)](http://cpdb.molgen.mpg.de/CPDB/showSetDetails?sp=p&st=2) | [2 (66.7%)](http://cpdb.molgen.mpg.de/CPDB/showSetDetails?sp=p&st=2) | 0.0145 | 0.191 | INOH |
| NF-kappa B signaling pathway | [95(10)](http://cpdb.molgen.mpg.de/CPDB/showSetDetails?sp=p&st=3) | [3 (30.0%)](http://cpdb.molgen.mpg.de/CPDB/showSetDetails?sp=p&st=3) | 0.0253 | 0.191 | KEGG |
| Glucocorticoid Receptor Pathway | [71(4)](http://cpdb.molgen.mpg.de/CPDB/showSetDetails?sp=p&st=4) | [2 (50.0%)](http://cpdb.molgen.mpg.de/CPDB/showSetDetails?sp=p&st=4) | 0.0279 | 0.191 | Wikipathways |
| TLR NFkB | [70(4)](http://cpdb.molgen.mpg.de/CPDB/showSetDetails?sp=p&st=5) | [2 (50.0%)](http://cpdb.molgen.mpg.de/CPDB/showSetDetails?sp=p&st=5) | 0.0279 | 0.191 | INOH |
| Nuclear Receptors Meta-Pathway | [316(12)](http://cpdb.molgen.mpg.de/CPDB/showSetDetails?sp=p&st=6) | [3 (25.0%)](http://cpdb.molgen.mpg.de/CPDB/showSetDetails?sp=p&st=6) | 0.0435 | 0.197 | Wikipathways |
| Type II interferon signaling (IFNG) | [37(5)](http://cpdb.molgen.mpg.de/CPDB/showSetDetails?sp=p&st=7) | [2 (40.0%)](http://cpdb.molgen.mpg.de/CPDB/showSetDetails?sp=p&st=7) | 0.0448 | 0.197 | Wikipathways |
|  |  |  |  |  |  |
| **Cluster 5** |  |  |  |  |  |
| Allograft Rejection | [80(14)](http://cpdb.molgen.mpg.de/CPDB/showSetDetails?sp=p&st=0) | [4 (28.6%)](http://cpdb.molgen.mpg.de/CPDB/showSetDetails?sp=p&st=0) | 0.00313 | 0.0532 | Wikipathways |
| Cytokine-cytokine receptor interaction | [265(25)](http://cpdb.molgen.mpg.de/CPDB/showSetDetails?sp=p&st=1) | [4 (16.0%)](http://cpdb.molgen.mpg.de/CPDB/showSetDetails?sp=p&st=1) | 0.0343 | 0.272 | KEGG |

*^a^ Set size = the total number of genes in each biological pathway. The number in brackets is the number of genes in that pathway that are contained in the background gene list.*

*^b^ Candidates = the number of genes in each pathway that are contained in the input gene list. The percentage in brackets is the number of candidates as a proportion of the number of genes present in the background gene list for each pathway.*

**Supplementary Table 2c. Differential regulation of clusters 1-5 in individuals with *C. trachomatis* infection and clinical signs.** A multivariable linear regression was performed using the first principle component as the combined cluster expression value for each individual. Using the Benjamini and Hochberg approach for adjusting for multiple comparisons, in order to control the false discovery rate <5% only tests with a p-value below 0.018 (highlighted in bold) are considered statistically significant.

|  | **Cluster 1** | | **Cluster 2** | | **Cluster 3** | | **Cluster 4** | | **Cluster 5** | |
| --- | --- | --- | --- | --- | --- | --- | --- | --- | --- | --- |
|  | P value | OR (CI) | P value | OR (CI) | P value | OR (CI) | P value | OR (CI) | P value | OR (CI) |
| **TS** | 0.063 | 0.63 0.38 - 1.03) | 0.334 | 1.24 (0.80 - 1.93) | 0.036 | 1.47 (1.03 - 2.10) | **0.014** | 1.75 (1.12 - 2.74) | **0.010** | 1.57 (1.12 - 2.22) |
| **TF** | 0.174 | 1.51 (0.84 - 2.72) | **0.016** | 1.91 (1.13 - 3.25) | 0.351 | 1.23 (0.80 - 1.88) | 0.370 | 0.78 (0.46 - 1.33) | 0.028 | 1.59 (1.05 - 2.40) |
| **TP** | 0.489 | 1.23 (0.69 - 2.20) | 0.044 | 1.71 (1.02 - 2.88) | 0.361 | 1.22 (0.80 - 1.86) | **3.94E-04** | 2.60 (1.54 - 4.40) | **2.53E-05** | 2.41 (1.61 - 3.62) |
| **Infection** | 0.590 | 1.19 (0.63 - 2.27) | **1.55E-15** | 11.29 (6.36 - 20.06) | **2.00E-16** | 17.05 (10.71 - 27.14) | 0.244 | 1.41 (0.79 - 2.52) | **1.23E-10** | 4.51 (2.88 - 7.05) |
| **Age** | 0.086 | 0.91 (0.82 - 1.01) | 0.111 | 0.92 (0.84 - 1.02) | **2.08E-06** | 0.82 (0.76 - 0.89) | **0.016** | 0.89 (0.80 - 0.98) | **7.53E-08** | 0.81 (0.75 - 0.87) |
| **Sex (F)** | 0.584 | 1.13 (0.74 - 1.72) | 0.081 | 1.40 (0.96 - 2.06) | 0.028 | 1.41 (1.04 - 1.92) | 0.724 | 0.93 (0.64 - 1.37) | 0.078 | 1.31 (0.97 - 1.76 |
