# Supplementary material for: Immunofibrogenic Gene Expression Patterns in Tanzanian Children with Ocular Chlamydia trachomatis Infection, Active Trachoma and Scarring: Baseline Results of a 4-Year Longitudinal Study
Source: Front Cell Infect Microbiol. 2017 Sep 15;7:406. doi: 10.3389/fcimb.2017.00406 (PMC5605569; doi:10.3389/fcimb.2017.00406)

Supplementary figure 1: Principal component analysis. (a) Infection and Active Trachoma status. (b) Scarring status.

(a)

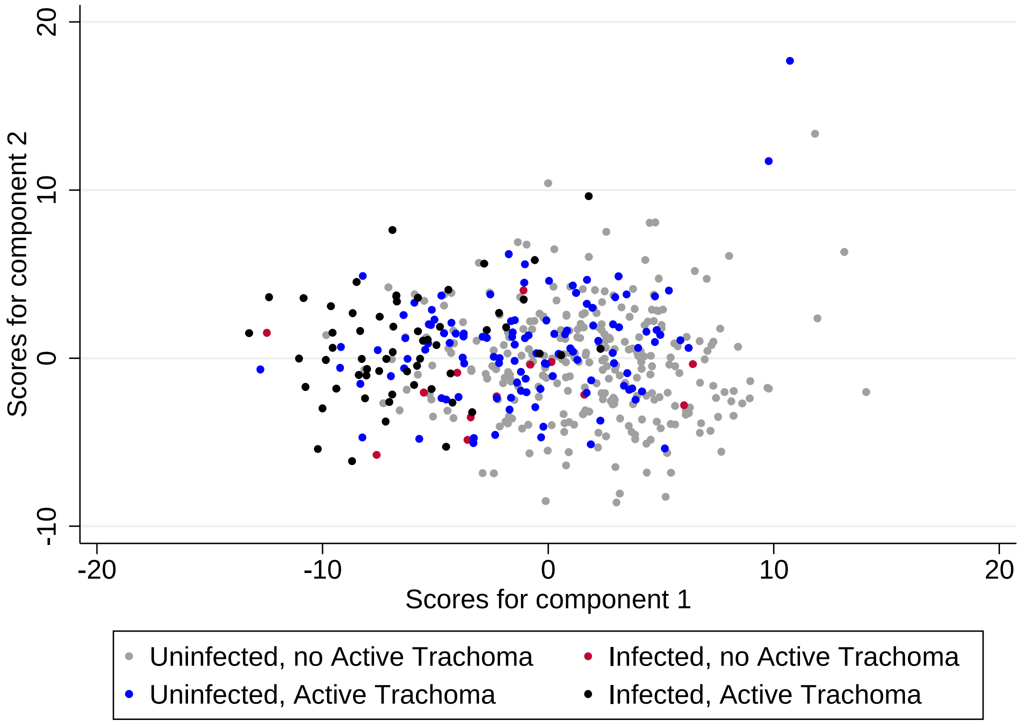

(b)

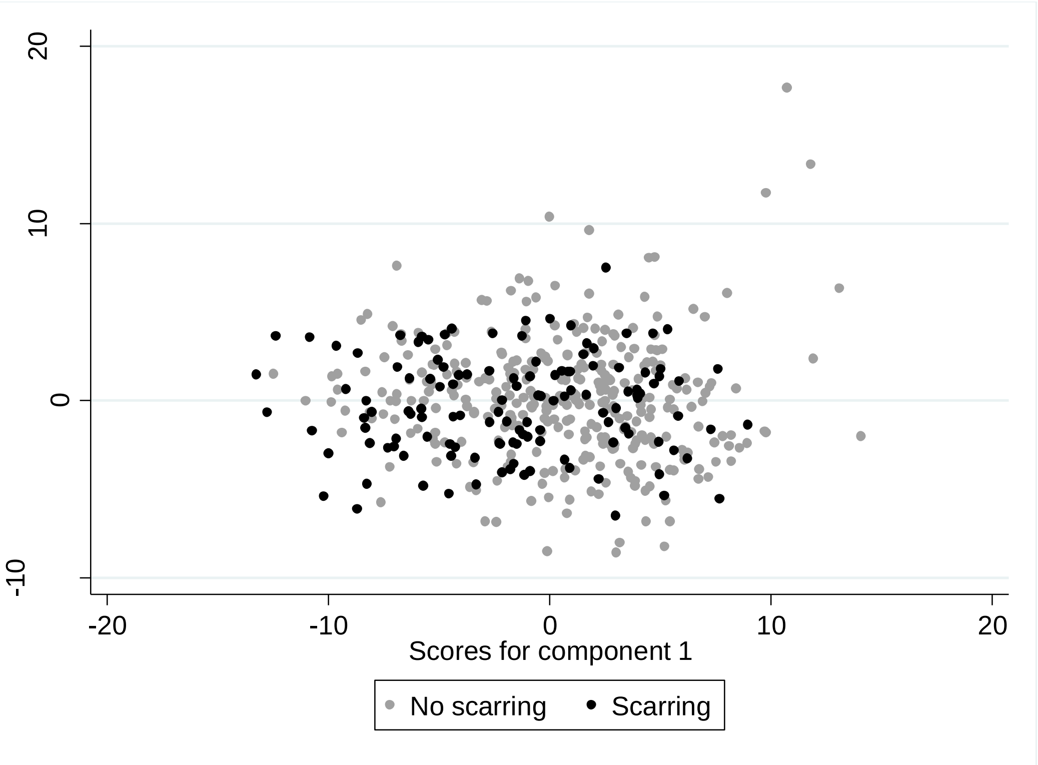

Supplement: Supplementary file 3 [file Image1.PDF]
